# Using biodiversity features to promote an Ecosystem-Based Conservation framework in terrestrial ecosystems

**DOI:** 10.1371/journal.pone.0336705

**Published:** 2025-11-21

**Authors:** Florence Godfrey Tarimo, Francis Moyo, Linus Kasian Munishi

**Affiliations:** 1 School of Life Sciences and Bio-Engineering, The Nelson Mandela African Institution of Science and Technology, Arusha, Tanzania; 2 Department of Geography and Economics, Mkwawa University College of Education (MUCE), Iringa, Tanzania; Instituto Federal de Educacao Ciencia e Tecnologia Goiano - Campus Urutai, BRAZIL

## Abstract

Biodiversity features are important indicators for monitoring conservation success and have been adopted to assess the extent of biodiversity loss in ecosystems affected by anthropogenic pressures. Protected areas (PAs) form the basis of the modern conservation framework and are the heart of conserving biodiversity. Despite PAs being the cornerstones for biodiversity conservation, they are limited in extent and their ability to inclusively conserve and safeguard biodiversity. We performed a literature review and spatial analysis across terrestrial ecosystems in Tanzania to investigate how biodiversity features can guide and promote an ecosystem-based conservation approach. We compiled biodiversity features for three taxonomic groups (plants, vertebrates, and invertebrates), resulting in a total sum of 22,987 features across eight terrestrial ecosystems in Tanzania. Our results revealed that the montane forest ecosystem had more total reported biodiversity features than other ecosystems, with <1/3 of its area protected. For other ecosystems, our three biodiversity features varied widely with the level of protection. PAs encompassed approximately 41%, while the remaining lacked formal protection measures. Invertebrates are often not considered when designing PA systems or building conservation approaches, highlighting the need to extend conservation efforts beyond the current framework. An ecosystem-based conservation framework is needed to extend conservation efforts outside PAs to address substantial taxonomic and spatial gaps in conservation actions to achieve biodiversity conservation targets under the Post-2020 Global Biodiversity Framework**.**

## Introduction

Protected areas (PAs) form the basis of modern conservation frameworks and are at the heart of conserving biodiversity [1,2]. Despite global efforts under the Aichi Targets, biodiversity loss continues, with wildlife populations declining by an average of over 50% [3]. In December 2022, the Conference of the Parties to the Convention on Biological Diversity (COP15) adopted the Kunming-Montreal Global Biodiversity Framework, which includes four global goals for 2050 and 23 targets aiming to achieve by 2030 [4–6]. This Framework calls for urgent action, including conserving areas vital for both biodiversity conservation and ecosystem services provision. Although PAs have been regarded as cornerstones for biodiversity conservation, mounting evidence shows that they are not immune to species loss, particularly in terms of vertebrate species [7]. Whilst evidence from around the world indicates that protected area (PA) networks are expanding, they often exclude ecologically important landscapes, limiting their ability to maintain intact natural ecosystems and/or achieve biodiversity conservation targets [8,9]. Regional frameworks, such as the Kigali Call to Action (2022), also emphasise the need to incorporate unprotected areas into conservation planning. These developments signal a shift towards more inclusive, ecosystem-level conservation approaches that could offer a better solution to improve the effectiveness of PAs in halting ongoing biodiversity loss [10–12]. A more inclusive conservation framework is, therefore, needed because current conservation strategies are unlikely to meet these global biodiversity commitments [13], underscoring the need for conservation approaches that extend beyond PAs, to effectively safeguard biodiversity right across dynamic social-ecological systems [14].

In Africa, conservation has evolved as a result of the intersection of ecological, cultural, institutional and economic factors [15,16]. Precolonial societies used customary laws to manage resources [17]. The colonial era imposed ‘preservationist’ model often disregarding indigenous knowledge [18,19]. Post-independence, maintained expansion of national parks often impacted local communities but by late 1990s, community-based natural resource management had emerged [20]. More recently CBD-inspired frameworks have promoted holistic approaches integrating social-ecological values and climate change adaptation measures [21–23].

Biodiversity conservation is recognised for both its intrinsic values, and the vital ecosystem services it offers to humanity [24,25]. Yet, challenges remain in implementing and sustaining effective conservation efforts, largely due to challenges in moving conservation beyond mere ‘protection’ into long-term rehabilitation and enhancement of ecosystems that can function and bring benefits beyond the limitations posed by PA boundaries. Biodiversity exists beyond formal boundaries and, as defined by CBD (1992), recognising and conserving ecosystem functions across spatial levels from species to landscapes, is critical for sustainability. In addition to these spatial challenges, existing conservation frameworks are often faced with incomplete data in terms of estimates of biodiversity features including species distributions at the ecosystem level. These challenges have received little academic attention to date, and it is imperative to address them by adopting the ecosystem level as the defined unit of analysis. What is needed is a holistic approach that acknowledges the interconnectedness of ecosystems and addresses biodiversity threats that exist within and beyond PA boundaries.

Ecosystem-based conservation has received considerable attention primarily through the Ecosystem-based Approach (EBA), established by the Convention on Biological Diversity [26,27]. The EBA is based on a framework that integrates the management of land, water, and living resources, recognising ecosystems as “dynamic complexes of plant, animal, and microorganism communities and their non-living environment interacting as a functional unit” [27]. However, EBA has rarely been operationalised for biodiversity assessment and planning. In this study, we propose an Ecosystem-Based Conservation (EBC) framework as a step forward on EBA, serving as an operational conservation strategy to increase conservation efforts beyond PAs. The EBC framework treats the ecosystems as social-ecological units, where native biota and human communities have co-evolved over time. These interactions have generated Traditional Ecological Knowledge (TEK), supporting coexistence and generating nature’s contributions to people through ecosystem service bundles [28]. In Tanzania, eight major terrestrial ecosystems exist, each with distinct biota and communities with unique TEK. These ecosystems and associated communities provide different opportunities for sustaining biodiversity conservation and provision of ecosystem services [29–31]. Unlike the PA approach, which confines conservation within boundaries, the EBC framework promotes biodiversity conservation as well as incentives for stewardship beyond the boundaries of PAs. The framework, therefore, offers a more inclusive biodiversity conservation strategy outside protected areas and a complementary strategy for effective biodiversity conservation at ecosystem level. Understanding biodiversity features that are unique to a specific ecosystem and how people and biodiversity overlap and interact outside protected area networks is crucial for establishing realistic, sustainable targets for guiding biodiversity conservation and ecosystem service delivery beyond protected areas. However, how unprotected areas of ecosystems and communities can sustain or enhance ecosystem services through improved conservation requires novel frameworks such as EBC that can guide biodiversity conservation and sustainable management at the ecosystem level; thus, optimising conservation efforts and benefits beyond protected areas.

Assessing biodiversity distribution across multiple ecosystems is crucial for informing ecosystem-based conservation strategies. Measuring and assessing aspects of biodiversity, even for well-studied taxa like birds and large mammals, however, remains challenging [32,33]; hence, biodiversity proxies are often used. In conservation science, biodiversity features such as species richness, indicator species, species-area curve, and biomass are commonly used proxies. Ecosystem services and bio-indicator species also help in the evaluation of ecosystem integrity [34,35]. In this study, biodiversity features refer to a group taxon level (i.e., key taxonomic groups, including vertebrates, plants, and invertebrates), that represent biological diversity within ecosystems. In many countries, including Tanzania, biodiversity data remain sparse and uneven, particularly outside protected areas and for less-studied taxa [36,37]. Literature-based data compilation is, therefore, a pragmatic method to estimate ecosystem-level biodiversity distribution [38]. We used the sum of reported biodiversity features in each of the three taxonomic groups as a proxy to estimate how biodiversity is distributed across different terrestrial ecosystems in Tanzania. While such data are shaped by sampling and publication biases, they still provide critical insights into underrepresented taxa and overlooked ecosystems. This approach aligns with recent Kunming-Montreal Global Biodiversity Framework targets [6,39] of supporting inclusive spatial management (Target 1); restoring degraded ecosystems (Target 2); increasing green and blue spaces in urban areas (Target 12), as well as evidence-based conservation planning.

Tanzania is a biodiversity-rich country that has expanded its PAs network, covering marine, terrestrial, and inland water areas, surpassing Global Biodiversity Framework Target 3 by protecting over 40% of its total area [40]. Despite these efforts, Tanzania, widely regarded as a key country for conservation in mainland Africa, is experiencing a rapid decline in biodiversity [41–43]. This loss is undermining ecosystem resilience and diminishing nature’s capacity to support human well-being [44–46]. Consequently, this study has focused on Tanzania to assess and delineate (i) terrestrial ecosystems found in Tanzania, (ii) PAs and their proportions across different ecosystems; and (iii) distribution estimates of biodiversity features in each ecosystem, as a proxy for species richness. The resulting data provide a foundation for developing and promoting a new and effective EBC framework, as a complementary strategy to enhance biodiversity conservation beyond PA boundaries.

## Methods

### Study area

**Fig 1:**
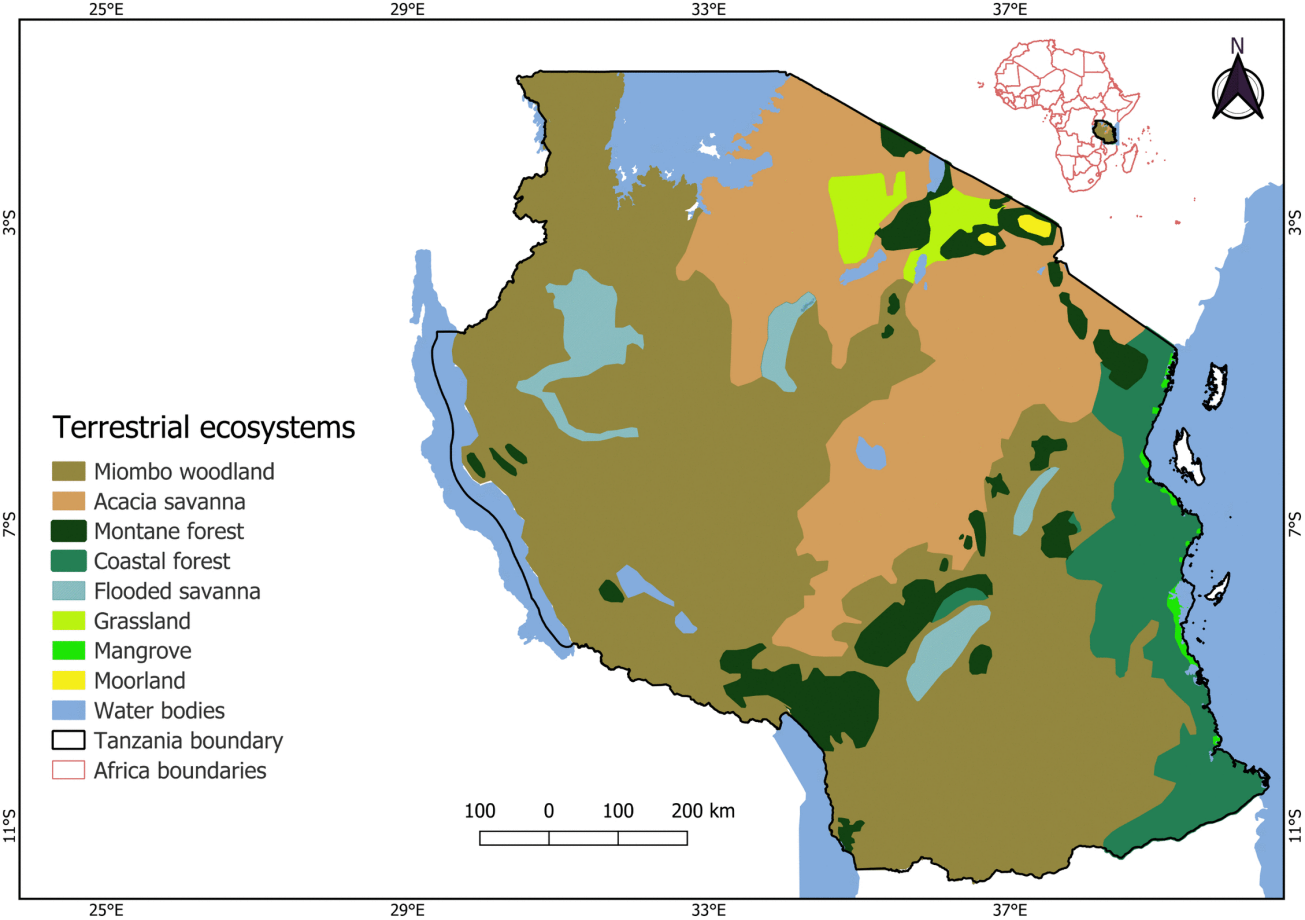
Major terrestrial ecosystems in Tanzania.

This study was conducted in eight major terrestrial ecosystems (Fig 1) on the mainland of Tanzania. Tanzania has an exceptionally high diversity of terrestrial ecoregions and biomes within the Afrotropical realm, making it one of the most ecologically diverse countries in Africa [47]. Tanzania is located between 29⁰E and 41⁰E and 1⁰S and 12⁰S, covering 945,100 km² and is endowed with a rich variety of landscapes from coastal plains, upland areas and mountains including Mount Kilimanjaro, Africa’s highest peak, at 5,895 meters above sea level. The country has various bioclimatic and topographic zones, ranging from dry regions with precipitation levels below 400 mm to humid regions with precipitation levels exceeding 2000 mm annually [48]. Across these zones, temperatures vary broadly, typically ranging from 5.3⁰C to 33.1⁰C [49]. The country’s climate is tropical and divided into four main climatic zones: hot humid coastal plain, semi-arid central plateau, high rainfall lake regions, and the temperate highlands [50].

### Classification of ecosystems

A map of terrestrial ecosystems in Tanzania was created using the shapefiles of Terrestrial Ecoregions of the World [47], that are available under a CC BY 4.0 licence. These shapefiles were clipped to Tanzania’s national boundaries and reclassified into ecosystem types following the UNEP [51] classes. All spatial operations, including clipping, reclassification, and visualisation, were conducted in QGIS version 3.38.0. The resulting map depicts eight types; grasslands, miombo woodland, acacia savanna, coastal forest, mangrove, moorland, montane forest, and flooded savanna (Fig 1).

### Delineating PAs and their proportions in different ecosystems

PAs are clearly defined geographical spaces, recognised, dedicated, and managed, through legal or other effective means, to achieve the long-term conservation of nature and associated ecosystem services and cultural values as defined by the IUCN [52]. For our study, we used three categories of land use within ecosystems: 1) Priority protected areas (PPAs) which included National parks, Ngorongoro conservation area, Nature reserves, Game reserves, and Forest reserves; 2) other protected areas (OPAs) which included Wildlife Management Areas, Game Controlled Areas and Game Open Areas; and 3) unprotected areas (UAs) which included included settlements, farmlands and pastures. PPAs differ from OPAs based on the level of human intervention allowed. In PPAs, human activities are either prohibited (e.g., nature reserves, national parks) or very limited (e.g., only sport hunting in game reserves). OPAs, on the other hand, allow human activities such as settlements, cultivation, and livestock keeping, while focusing on the sustainable use of natural resources. We analysed PA coverage across different ecosystem types using a spatial dataset from the Tanzania Wildlife Research Institute (TAWIRI). We used the QGIS intersect tool to determine the area of each ecosystem type within each category (Fig 3). We also determined the number of PAs within each ecosystem type, and for any PA that extended beyond one ecosystem type, its count was assigned to the ecosystem where the largest portion of its area was located.

**Fig 2:**
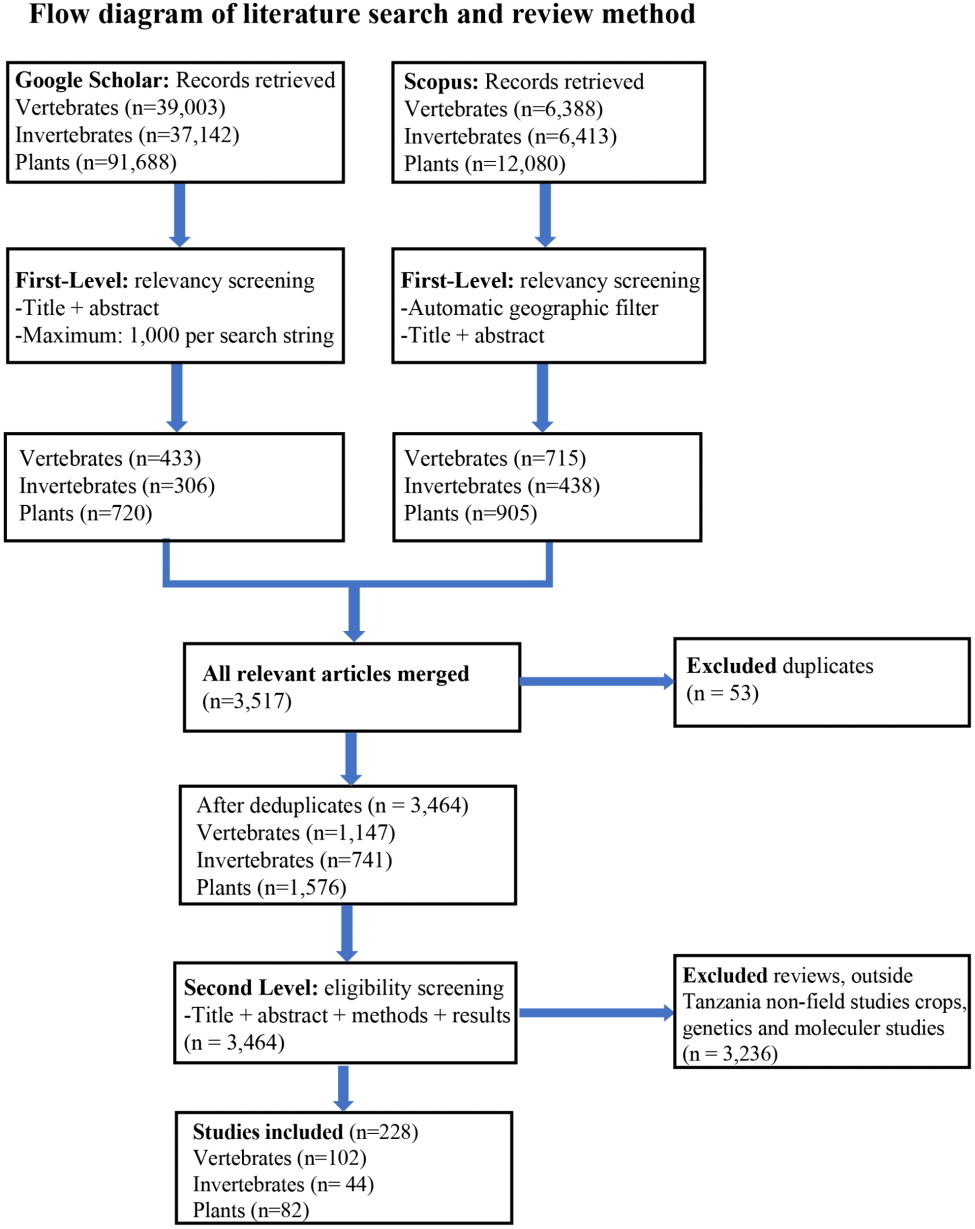
Flow diagram of literature search and review method.

### Establishing distribution estimates of biodiversity features

As a consequence of a general lack of biodiversity information at ecosystem level, we derived a pragmatic approach for obtaining distribution estimates of three biodiversity features across different ecosystems, to assess progress towards achieving the Kunming-Montreal Global Biodiversity Framework goals. We assessed biodiversity distribution by conducting a literature search of biodiversity occurrence within Tanzania’s major terrestrial ecosystems, focused on three biodiversity features (vertebrates, invertebrates, and plants), which are the most studied in terrestrial ecosystems [34]. Ecosystem boundaries guided this as our primary unit of analysis.

We conducted a comprehensive literature search in Scopus and Google Scholar databases from May 2023 to August 2023. These databases were chosen for their ease of access and extensive coverage. In both databases, results were restricted to peer-reviewed articles, sorted by relevance, and without time limitation. We utilised tailored search strings that were designed to be ecosystem- and taxon-specific, combining habitat descriptors with biodiversity and taxonomic keywords using Boolean operators (AND, OR). The complete set of search strings is included in the supplementary materials (S1 Table). Each search string was applied in each database separately for vertebrates, invertebrates, and plants within a given ecosystem, and the number of articles retrieved was recorded per database, per ecosystem and per taxon. In Google Scholar, we screened abstracts of up to 1,000 articles per search string, due to Google Scholar platform constraints, whereas in Scopus, all retrieved articles were screened. The search date and the number of articles retrieved were documented for each ecosystem-taxon combination.

The first-level screening assessed titles and abstracts when necessary to determine whether each study was biodiversity-related and conducted in Tanzania. In Scopus, the geographic filter automatically excluded non-Tanzanian studies, while biodiversity relevance was assessed manually. In Google Scholar, both biodiversity relevance and geographic scope were determined manually from titles and abstracts. Studies were excluded at this stage only if they were clearly irrelevant to biodiversity or explicitly indicated a study area outside Tanzania. For each database, the number of studies identified as relevant after screening was recorded by taxon and ecosystem. All Studies retained after the first-level screening were merged across databases by taxon and ecosystem, and duplicates were eliminated so that each study was only counted once in the aggregation (Fig 2).

At the second-level screening, we reviewed the title, abstract, methods, and results sections, while the introduction and discussion sections were not considered. Each study was initially assigned to a terrestrial ecosystem based on the search string under which it was retrieved. If the reported study area did not match the ecosystem implied by the search string, the study was reassigned. Final assignments were verified using the study’s area description (e.g., habitat, geographic location, coordinates, or maps) provided in the methods or results sections. Full eligibility criteria were then applied; studies were included if they reported field-based primary data, presented in absolute numbers on biodiversity distribution or species richness within Tanzania’s terrestrial ecosystems. Studies were excluded if they were conducted outside Tanzania, focused on aquatic systems or crops, relied solely on molecular/genetic approaches or modelling without field data, or presented non-primary literature such as reviews (Fig 2).

From the final pool of eligible studies, biodiversity data were extracted in a standardised manner. When article reported aggregated counts of species (e.g., insects, snails, birds, reptiles, amphibians, trees, bryophytes), these were summed within the appropriate biodiversity feature category: vertebrates, invertebrates, or plants. When species-level inventories were provided, species were counted individually, and the total count was assigned to their respective taxonomic group. Therefore, the number of biodiversity features reported in this study represents the aggregate totals of species within the three key taxonomic groups (plants, vertebrates, and invertebrates) as presented in each reviewed article, rather than unique species inventories. The number of articles contributing data for each taxon is summarised in Supplementary Materials (S1 Table).

For each study, we documented the total counts of biodiversity features reported within each taxon and ecosystem. These counts, whether presented as individual species or aggregated groups (e.g., number of bird species), were summed across all included studies to obtain totals per taxonomic group and ecosystem. The specific counts extracted from each article are provided in the supplementary materials (S2 Data). The combined sum of reported biodiversity features from all three taxonomic groups within each ecosystem was expressed as a number per 100 km^2^ [53]. In addition, the summed totals for each taxonomic group within each ecosystem were converted into percentages to enable comparisons across taxa and ecosystems.

While biodiversity is inherently complex and difficult to quantify precisely, our approach provides a practical framework for ecosystem-level assessment. We adopted the CBD (1992) definition of biodiversity, which encompasses diversity within species, between species and of ecosystems, allowing us to capture representative information necessary for developing and improving conservation strategies at ecosystem-scale. The articles that were reviewed were used to estimate the presence or absence of key biodiversity features within each ecosystem. Although the exact locations of individual species were not specified in the studies, the species were assumed to occur across various habitat areas within the respective ecosystem. Whilst this approach does not provide exact estimates of species richness, it serves as a pragmatic proxy for assessing biodiversity distribution at ecosystem-level.

## Results

### Protected and unprotected areas in different ecosystems

**Fig 3:**
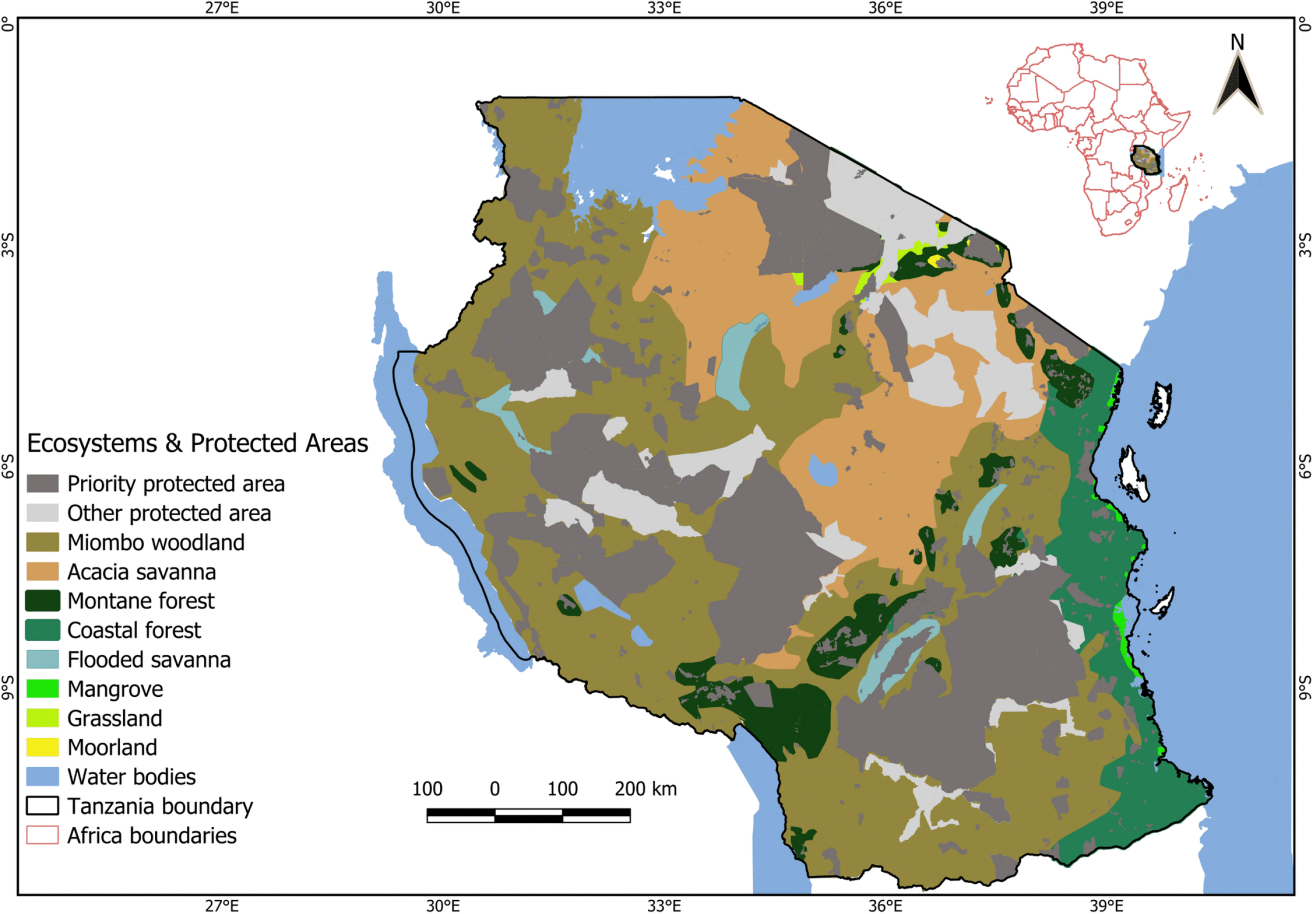
Extent of PA coverage across different terrestrial ecosystems in Tanzania.

The scale of Tanzania’s terrestrial ecosystems are summarised along with the proportions of protected and unprotected areas within them (Table 1; Figs 1 and 3). Miombo woodland was the largest, covering 484,942 km² (55%), while moorland being the smallest, covering 1,426 km² (0.2%) (Fig 1). The total amount of protected land varied, with protected areas (PPAs and OPAs) covering 41% (358,528 km²) of the land, with the remaining 59% (523,361 km²) being unprotected (outside formal designations). In grassland ecosystem, 89.2% of the land area was protected, whilst for mangrove ecosystem only 8% were under protection. For montane forest ecosystem less than a third of the total area was protected. Over half of the area in most ecosystems lacked protection with coastal, and montane forests particularly vulnerable with 77% and 70% of their total areas respectively unprotected (Table 1). Fig 3 shows the proportions of these ecosystems that were protected under PPA and OPA categories.

**Table 1:** Terrestrial ecosystems in Tanzania, their scale and levels of protection.

Ecosystem type	Ecosystem size (km^2^)	PPAs^a^ (%)	OPAs^b^ (%)	UAs^c^ (%)	Number of PAs
Acacia savanna	212,050	20	15	65	PPAs 100, OPAs 12
Flooded savanna	32,680	48	7	45	PPAs 13, OPAs 1
Grassland	17,727	54	35	11	PPAs 7, OPAs 2
Coastal forest	69,624	21	2	77	PPAs 84, OPAs 2
Montane forest	58,356	23	7	70	PPAs 194, OPAs 1
Mangrove	5,084	8	0	92	PPAs 9, OPAs 0
Miombo woodland	484,942	32	12	56	PPAs 232, OPAs 19
Moorland	1,426	80	0	20	PPAs 1, OPAs 0

a Priority protected areas

b Other protected areas

c Unprotected areas

PPAs covered 29% (254,632 km²) of the total terrestrial land area within Tanzania whilst OPAs covered an additional 12% (103,897 km²). PPAs covered 80% of the moorland and 54% of grassland ecosystems, respectively. In mangrove ecosystem, PPAs covered only 8% of its total area. In contrast, 32% of miombo woodland ecosystem were under PPAs designations, with an additional 12% under OPAs designations (Table 1). The total number of PPAs was 640, while the number of OPAs was 37. Miombo woodland and montane forest ecosystems were covered by the most PPAs with 232 and 194 respectively, whilst moorland was only covered by one PPA (Table 1). In terms of OPAs, miombo woodland had the highest number [19], with mangrove ecosystem completely lacking any designated OPAs (Table 1).

### Distribution and abundance estimate of biodiversity features

The literature search yielded a total of 3,464 published articles, after removal of duplicates, that were initially considered relevant to the study objective. Of these, 1,576 related to plant species; 1,147 related to vertebrates; and 741 related to invertebrates (Fig 2). Following application of inclusion and exclusion criteria, a total of 228 articles were selected for data extraction, including 102 articles for vertebrates, 82 for plants, and 44 for invertebrates (Fig 2). From these articles, 22,987 biodiversity feature estimates were included from eight terrestrial ecosystems in Tanzania. Of the 22,987 biodiversity features, plants comprised 12,595 counts; vertebrates accounted for 5,963 counts; and invertebrates accounted for 4,429 counts (see S2 Data). These figures are the sum total of biodiversity features reported in each ecosystem category and not unique species.

Across ecosystem categories, the montane forest stood out with the highest number of reported biodiversity features at 18 per 100 km², followed by coastal forest and grassland ecosystems, which both had eight features per 100 km². In contrast, acacia savanna, flooded savanna, and miombo woodland ecosystems each had just one biodiversity feature per 100 km² (Table 2).

**Table 2:** Biodiversity features and their estimates per 100 km^2^ as drawn from the literature.

Ecosystem type	Vertebrates	Invertebrates	Plants	Total species/100 km²
**Acacia savanna**	218	1472	481	1
**Flooded savanna**	73	0	230	1
**Grassland**	365	512	522	8
**Coastal forest**	2,176	189	3235	8
**Montane Forest**	1,874	1,600	70,53	18
**Mangrove**	64	52	61	3
**Miombo woodland**	1169	604	1008	1
**Moorland**	24	0	5	2

**Fig 4:**
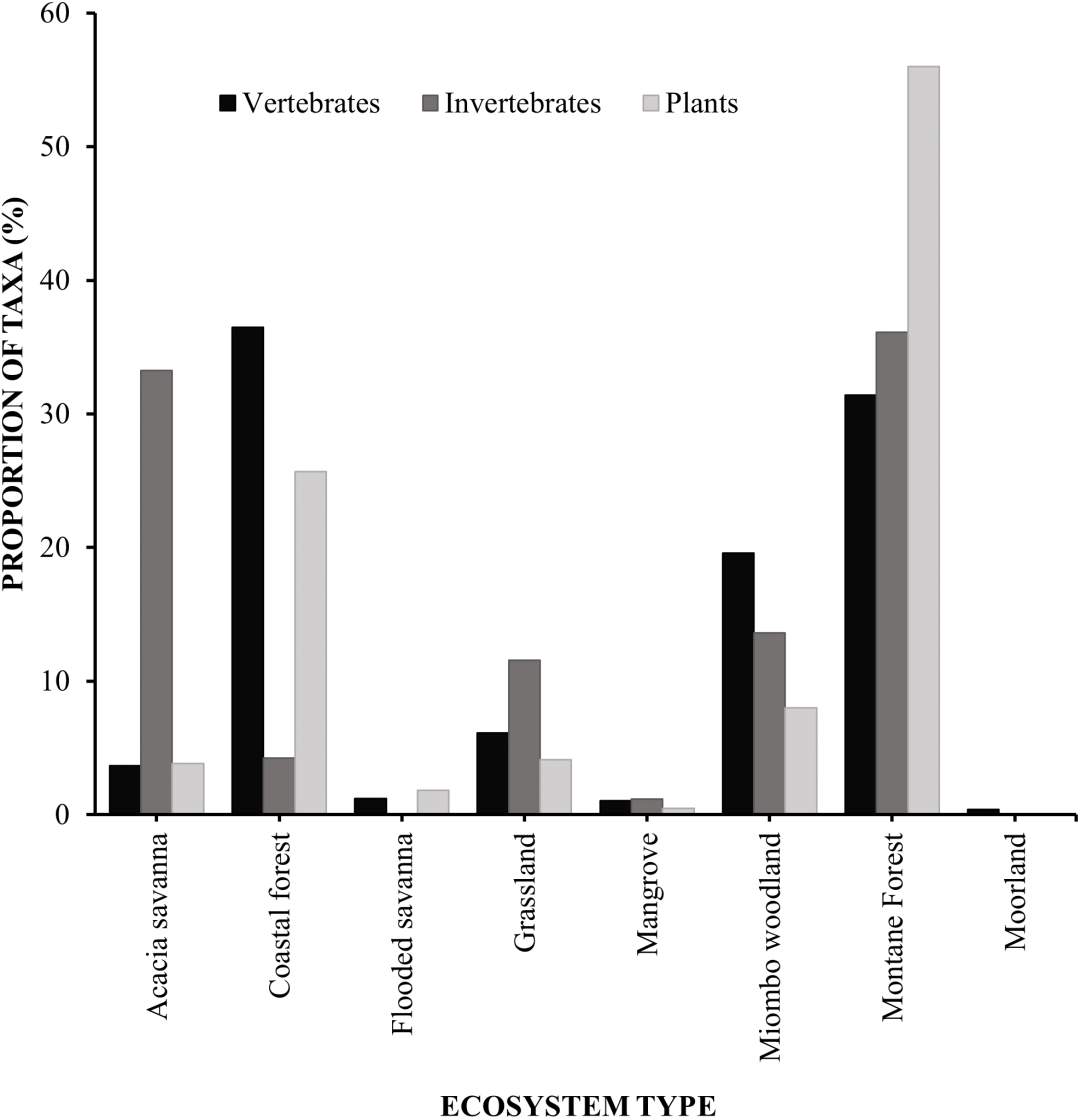
Proportions of biodiversity features estimated in various Tanzanian ecosystems, based on the literature search.

Reported biodiversity features varied considerably between ecosystems and taxonomic groups. Based on the reviewed literature, montane forest ecosystem had the highest biodiversity features totals across all three taxa, with 7,053 plant records (56.0%); 1,600 invertebrate records (36.1%); and 1,874 vertebrate records (31.4%). Coastal forest ecosystem ranked second, with 3,235 plant records (25.7%); 2,176 vertebrate records (36.5%); and 189 invertebrate records (4.3%). In contrast, moorland ecosystem had the lowest number with only 24 vertebrate records (0.4%) and 5 plant records (0.04%) (Table 2; Fig 4).

## Discussion

Unprotected areas within terrestrial ecosystems tend to receive less scientific attention [54,55] and are often overlooked in conservation frameworks. As a result, they can often face increasing threats from human activities. This study used literature-based species occurrence data to provide a broad assessment of biodiversity distribution across Tanzania’s terrestrial ecosystems. Given that conserving global biodiversity is a critical challenge, particularly in the face of a changing climate and increasing anthropogenic pressures, the lack of protection for fragile ecosystems is worrying [56–58]. Understanding the distribution of biodiversity features and the extent to which they are conserved in different ecosystems is vital for evidence-based, effective conservation planning. In regions where field-based inventories are limited or unavailable, such literature-derived estimates can serve as a practical proxy for assessing biodiversity distribution at the ecosystem scale and informing conservation planning. The results generated in this study contain information that is essential for effective conservation planning and for developing a robust, ecosystem-based conservation approach that can support efforts to halt biodiversity loss. The sum of biodiversity features estimated here can be extrapolated to highlight species abundance at ecosystem level, given that the method is based on abundance and diversity studies in similar ecosystems (e.g., tropical forests hosting over 60% of global terrestrial vertebrates and comprising a substantial portion of global biodiversity) [59]. The majority of the biodiversity taxa in Tanzania, in non-protected areas of these species-rich, diverse ecosystems, have received little conservation attention. This suggests many species are inadequately protected, given that the proportion of unprotected areas within these ecosystems ranges from 11% in the most protected to 92% in the least protected. These results should be useful not only to national and international decision-makers, but to everyone with a stake in Tanzania’s diverse ecosystems [60]. In order to address the current gaps in awareness, conservation protection and formal designation, it is crucial to adopt the EBC framework that encompasses whole ecosystems, thus broadening the current but fragmented protected area approach to biodiversity conservation. This EBC framework is particularly timely as conservation efforts intensify in response to biodiversity loss, alongside recognition of the need for conservation actions to incorporate multiple knowledge types and stakeholder groups, to achieve balanced environmental as well as socio-economic and cultural outcomes.

### Level of protection versus biodiversity conservation

In common with Brazil and Venezuela, Tanzania has successfully designated more than 39% of its terrestrial land [61,62] as PAs [40], exceeding conservation targets [63]. This includes PAs, such as National Parks, Conservation Areas, Nature Reserves, Forest Reserves, Forest Nature Reserves, Game Reserves, Marine Parks and Reserves, Game-Controlled Areas, Open Game Areas, and Wildlife Management Areas [40,63]. Despite the proportion of designated PAs in Tanzania exceeding the Global Biodiversity Framework Target 3 [6], our findings show that more than 50% of the majority of terrestrial ecosystems in Tanzania lack formal protection. Most conservation efforts currently tend to focus on the protection of biodiversity found within PAs, even though significant biodiversity is known to occur beyond these areas [64]. For example, 56% of Wildebeest movements occurred outside of the protection of Tarangire, Manyara, and Lake Natron National Parks [65]; and 10–20% of Tanzania tree species are found outside PAs [66]. Whilst the PA-oriented conservation approach is long recognized as a cornerstone in safeguarding biodiversity [67], its effectiveness in halting ongoing biodiversity loss is limited [7]. Amongst the reasons for this is a prevalent misconception surrounding conservation frameworks, where the term “conservation” is often erroneously equated with “protection”. This misinterpretation implies that conservation efforts should mostly focused on PAs. To address this gap, an ecosystem-based conservation framework is urgently needed to ensure biodiversity conservation is properly integrated into broader social-ecological landscapes management, processes that extend well beyond PAs [68,69].

### Distribution and abundance estimates of biodiversity features and levels of protection

Our results show indicative estimates of the distribution of biodiversity features in Tanzanian ecosystems. Montane forest ecosystem had the highest sum total of reported biodiversity features, reflecting its important role as a biodiversity hotspot and a refuge for many endemic species, particularly closed-forest specialists [70]. In contrast, other ecosystems including miombo woodland and savannas, had a lower sum total of biodiversity features, suggesting spatial heterogeneity in biodiversity distribution.

Tanzania has 677 PAs, with over 90% classified as PPAs. However, the distribution of PAs is uneven, and a high number of PAs does not necessarily align with areas of high biodiversity richness, or the presence of key endemics. Many ecosystems with important biodiversity features remain underrepresented within the PA network. For instance, montane forests harbour endemic species that are increasingly threatened by forest loss and fragmentation, reducing habitat quality and connectivity, and posing further challenges for their long-term survival, yet 70% of this ecosystem remains outside of formal protection designations in Tanzania.

These patterns highlight the limitations of relying solely on PA coverage for effective biodiversity conservation. The EBC framework, by contrast, can complement the PA network by addressing gaps in conservation, promoting landscape-scale connectivity, and facilitating participatory restoration and conservation efforts. Adopting EBC strategies in underrepresented ecosystems provides important opportunities to enhance the resilience and sustainability of biodiversity conservation efforts across terrestrial ecosystems.

### Implications of PA approach on conservation and management of biodiversity

Despite surpassing the global target for PAs as set by the CBD (2010), Tanzania ranks 11^th^ globally and 2^nd^ in Africa (behind Madagascar) for the highest number of threatened species on the IUCN Red List (2023). This includes nearly 200 native vertebrate species, 137 invertebrate species, and 1,031 plant species, which are classified as threatened, vulnerable, endangered, or critically endangered (IUCN, 2023). These figures highlight that broad PA coverage alone is not enough to prevent high levels of biodiversity loss. Consistent with this, our findings have shown that a significant proportion of most Tanzanian ecosystems remained unprotected, indicating that key biodiversity areas are potentially at risk. Furthermore, invertebrate taxa were reported in fewer studies compared with other taxonomic groups (see S2 Data). This aligns with broader evidence that biodiversity monitoring and conservation planning often show taxonomic bias, with PAs disproportionately focused on certain taxa, while neglecting others [71]. For instance, Casanelles‐Abella, Fontana [72] reported a spatial mismatch between invertebrate hotspots and PAs, while Wu, Yu [73] showed that the existing network of PAs in southwest China has less than a quarter of overlap with conservation hotspots, and only a fraction of these hotspots receive adequate protection. Taken together, these results emphasize that PAs as a conservation approach often fail to address crucial ecological processes such as individual home ranges and propagule dispersal [74], suggesting that relying solely on PAs is inadequate for fully understanding biodiversity distribution and abundance patterns across taxa and ecosystems.

Similar limitations have been documented across East Africa, where PAs cover just 10% of the geographic range of endemic/near-endemic vertebrate species, and only 26% of endemic species have at least half of their range protected [75]. Such limited spatial coverage undermines the effectiveness of PAs in conserving biodiversity and exacerbates risks from habitat loss and fragmentation, particularly for wide-ranging and long-distance dispersal species [76] under accelerating climate change [77]. Williams, Rondinini [78] have further demonstrated that many mammal species remain without effective population-level protection, especially in tropical biodiversity hotspots where PA coverage is minimal.

The concentration of conservation efforts only within PAs also compromises habitat connectivity. In Tanzania, about half of PAs remain connected, while many forest reserves exist as isolated fragments [63,79,80]. Wildlife corridors that are essential for maintaining ecological connectivity between PAs have declined in both number and functional integrity due to increasing land-use change and human encroachment [81]. For instance, in the Tarangire-Manyara ecosystems [65], and in the Kilombero valley [79], key corridors have experienced severe degradation. Kitendeni wildlife corridor in northern Tanzania, once 10 km wide [82], has narrowed to 6 km [83]. Such losses mirror a global trend where migratory routes are increasingly overlooked in conservation planning, leading to widespread disruption of animal movements [84,85]. Collectively, these patterns underscore the urgent need for conservation approaches that extend beyond PA boundaries and reinstate ecosystem-scale connectivity to enhance biodiversity resilience and the long-term sustainability of conservation efforts.

### The need for an Ecosystem-Based Conservation framework

While the international commitment to protect 30% of the world’s surface by 2030 is necessary, achieving meaningful biodiversity conservation requires inclusive and elevated conservation approaches that extend beyond PAs. Despite wide agreement that PPAs (IUCN category I-III) are the best approach for conserving biodiversity, they are limited in extent and exclude many species of key conservation importance [86]. In Africa, extending conservation practices beyond PAs remains underutilised, despite its potential to safeguard diverse taxa [87]. Our results indicate that a large proportion of Tanzania’s ecosystems remain unprotected, reinforcing the need for ecosystem-scale approaches that can complement formal PAs.

Rapid human population growth in Africa is projected to increase fivefold by the year 2100 [88,89], exacerbating pressures on ecosystems. OPAs (IUCN category IV-VI), which comprise over 60% of the global protected-area network, are often undervalued for biodiversity conservation in Africa due to their limited populations of large charismatic mammals [86]. Similarly, unprotected areas of ecosystems remained overlooked, as conservation frameworks and funding continue to prioritise PAs, assuming that they are the most cost-effective and logistically feasible option (Cuthbert et al., 2022; Gallardo et al., 2022). Yet evidence shows that substantial biodiversity exists outside PA boundaries, and in some cases even exceeds that within [90]. This highlights the need of complementary conservation approaches at ecosystem scale.

The EBC framework can address these gaps by treating ecosystems as social-ecological units, where native biota and human communities have co-evolved over time, generating traditional ecological knowledge that underpins both coexistence and resource use. By linking biodiversity features with ecosystem service bundles, the EBC framework provides a mechanism to incentivise communities to engage in conservation beyond PAs. For instance, in Tanzania’s montane forest ecosystem, bundles of services such as water provision, carbon storage, and ecotourism have been demonstrated [29–31], while in savanna rangelands, fodder and grazing resources play a critical role [91]. The generation of these ecosystem service bundles shows how sustaining native biota in these ecosystems generates multiple benefits that can continuously support human well-being and biodiversity simultaneously. Importantly, the EBC framework remains highly relevant for Tanzania, where PA coverage already exceeds global recommendations but biodiversity loss continues. This paradox reflects the fact that biodiversity does not follow administrative or PA boundaries.

By building on TEK and the incentives provided by ecosystem service bundles, EBC can mobilise local stewardship, promote sustainable use, and help reverse biodiversity loss, particularly in unprotected areas of these ecosystems. In Tanzania, different ecosystems are inhabited by diverse ethnic communities that interact with biodiversity features, particularly plant taxa, which are unique to their respective ecosystems and provide multiple values and ecosystem services. Among biodiversity features of these terrestrial ecosystems, some plant species occur in multiple locations and are known by different vernacular names and primary uses [92]. Of the various known plant species in these ecosystems, most are used for food, while others serve medicinal, material, or ritual purposes. The EBC framework can serve as an operational and inclusive strategy to incentivise communities to actively conserve plant species that sustain their livelihoods and support their well-being in these ecosystems in Tanzania and beyond.

By encompassing both formal PAs and their contiguous surroundings, the EBC framework aligns with IUCN connectivity conservation guidelines [69] and safeguards biodiversity, cultural values, and traditional resource management systems, particularly those practiced by indigenous and local communities. This way, EBC provides not only a framework to expand conservation beyond PAs but also a pathway for equitable cost-benefit sharing between communities and ecosystems. Implementing this framework requires context-specific prioritisation, ecosystem-level biodiversity monitoring, and engagement with communities who hold the knowledge and incentives to sustain biodiversity over the long term. Positioned alongside PAs, the EBC framework can enhance the resilience of ecosystems, maintain nature’s contributions to people, and ensure that conservation efforts respond to both ecological and social realities.

Although advances in connectivity science, biodiversity data availability, and computational modelling now enable more sophisticated assessments of functional diversity [93–97], keeping track of these advances is challenging for practitioners and stakeholders. Our study relies on literature-based biodiversity features as proxies for species distribution estimates. Accordingly, our analysis focuses on the presence or absence of key biodiversity features rather than unique species, since not all studies reported individual species. To allow comparisons across ecosystems, combined totals were standardised per 100 km²; however, these values should be interpreted as indicative of biodiversity representation rather than absolute measures. While this approach is constrained by uneven research effort, publication bias, and unequal ecosystem and taxon representation, it provides a reasonable estimate of the distribution of the three key biodiversity features across ecosystems [96,97]. Whilst PAs remain crucial for biodiversity conservation, our study has demonstrated that effective biodiversity conservation requires a broader ecosystem-level framework engaging all stakeholders. Implementing the EBC framework alongside PAs can help to better address biodiversity loss, particularly outside PAs, and enhance the long-term resilience of both ecosystems and human communities. Conservation or restoration of multiple patches of ecosystems is critical, particularly in fragmented habitats, and future studies should prioritise ecosystem-level biodiversity inventories comparing PAs and areas outside of PAs, to better understand variations in species abundance and diversity.

## Supporting information

S1 Table Summary of literature search results for taxa-specific biodiversity studies across eight terrestrial ecosystems in Tanzania. This table presents search terms, databases (Google Scholar and Scopus), total articles retrieved, relevant articles identified, and those included for data extraction for vertebrates, invertebrates, and plants across each ecosystem type.
(PDF)

S2 Data Compiled dataset of biodiversity records extracted from published studies across Tanzania’s eight terrestrial ecosystems. The dataset summarises the sum of taxonomic group (vertebrates, invertebrates, plants) species counts, and their corresponding references.
(PDF)

## References

[1] Pecl GT, Araújo MB, Bell JD, Blanchard J, Bonebrake TC, Chen I-C, et al. Biodiversity redistribution under climate change: Impacts on ecosystems and human well-being. Science. 2017;355(6332):eaai9214. doi: 10.1126/science.aai9214 28360268

[2] Dinerstein E, Vynne C, Sala E, Joshi AR, Fernando S, Lovejoy TE, et al. A Global Deal For Nature: Guiding principles, milestones, and targets. Sci Adv. 2019;5(4):eaaw2869. doi: 10.1126/sciadv.aaw2869 31016243 PMC6474764

[3] Westveer J, Freeman R, McRae L, Marconi V, Almond R, Grooten M. A deep dive into the living planet index: A technical report. Gland, Switzerland: WWF. 2022.

[4] Carroll C, Hoban S, Ray JC. Lessons from COP15 on effective scientific engagement in biodiversity policy processes. Conserv Biol. 2024;38(2):e14192. doi: 10.1111/cobi.14192 37768193

[5] Environment CC, Secretariat D. Post-2020 global biodiversity conservation. Green recovery with resilience and high quality development. Springer. 2023.

[6] Global Biodiversity Framework. Kunming-Montreal Global Biodiversity Framework. 2022.

[7] Craigie ID, Baillie JEM, Balmford A, Carbone C, Collen B, Green RE, et al. Large mammal population declines in Africa’s protected areas. Biological Conservation. 2010;143(9):2221–8. doi: 10.1016/j.biocon.2010.06.007

[8] Rodríguez-Rodríguez D, Martínez-Vega J. Effectiveness of Protected Areas in Conserving Biodiversity: A Worldwide Review. Cham, Switzerland: Springer. 2022.

[9] Ivanova IM, Cook CN. The role of privately protected areas in achieving biodiversity representation within a national protected area network. Conservat Sci and Prac. 2020;2(12). doi: 10.1111/csp2.307

[10] Bhola N, Klimmek H, Kingston N, Burgess ND, van Soesbergen A, Corrigan C, et al. Perspectives on area-based conservation and its meaning for future biodiversity policy. Conserv Biol. 2021;35(1):168–78. doi: 10.1111/cobi.13509 32277780 PMC7984296

[11] Hermoso V, Carvalho SB, Giakoumi S, Goldsborough D, Katsanevakis S, Leontiou S, et al. The EU Biodiversity Strategy for 2030: Opportunities and challenges on the path towards biodiversity recovery. Environmental Science & Policy. 2022;127:263–71. doi: 10.1016/j.envsci.2021.10.028

[12] Maxwell SL, Cazalis V, Dudley N, Hoffmann M, Rodrigues ASL, Stolton S, et al. Area-based conservation in the twenty-first century. Nature. 2020;586(7828):217–27. doi: 10.1038/s41586-020-2773-z 33028996

[13] Hagerman SM, Pelai R. “As Far as Possible and as Appropriate”: Implementing the Aichi Biodiversity Targets. Conservation Letters. 2016;9(6):469–78. doi: 10.1111/conl.12290

[14] Pressey RL, Cabeza M, Watts ME, Cowling RM, Wilson KA. Conservation planning in a changing world. Trends Ecol Evol. 2007;22(11):583–92. doi: 10.1016/j.tree.2007.10.001 17981360

[15] Kelemu S, Niassy S, Torto B, Fiaboe K, Affognon H, Tonnang H, et al. African edible insects for food and feed: inventory, diversity, commonalities and contribution to food security. JIFF. 2015;1(2):103–19. doi: 10.3920/jiff2014.0016

[16] Lama WB, Sattar N. Mountain tourism and the conservation of biological and cultural diversity. Key issues for mountain areas. 2004. p. 111–48.

[17] Giacomini G. Participatory rights, conservation and indigenous customary law. Indigenous peoples and climate justice: A critical analysis of international human rights law and governance. Springer. 2022:227–314.

[18] Kelly C, Wynants M, Munishi LK, Nasseri M, Patrick A, Mtei KM, et al. ‘Mind the Gap’: Reconnecting Local Actions and Multi-Level Policies to Bridge the Governance Gap. An Example of Soil Erosion Action from East Africa. Land. 2020;9(10):352. doi: 10.3390/land9100352

[19] Rodrigue-Allouche S. Conservation and indigenous peoples: The adoption of the ecological noble savage discourse and its political consequences. 2015.

[20] Child B, Barnes G. The conceptual evolution and practice of community-based natural resource management in southern Africa: past, present and future. Envir Conserv. 2010;37(3):283–95. doi: 10.1017/s0376892910000512

[21] Corson C, Gruby R, Witter R, Hagerman S, Suarez D, Greenberg S, et al. Everyone′s Solution? Defining and Redefining Protected Areas at the Convention on Biological Diversity. Conservat Soc. 2014;12(2):190. doi: 10.4103/0972-4923.138421

[22] Schmitz OJ, Lawler JJ, Beier P, Groves C, Knight G, Boyce DA Jr, et al. Conserving Biodiversity: Practical Guidance about Climate Change Adaptation Approaches in Support of Land-use Planning. Natural Areas Journal. 2015;35(1):190–203. doi: 10.3375/043.035.0120

[23] IPBES. Summary for policymakers of the global assessment report on biodiversity and ecosystem services of the Intergovernmental Science-Policy Platform on Biodiversity and Ecosystem Services. Bonn, Germany: Intergovernmental Science-Policy Platform on Biodiversity and Ecosystem Services. 2019.

[24] Baciu GE, Dobrotă CE, Apostol EN. Valuing forest ecosystem services. Why is an integrative approach needed?. Forests. 2021;12(6):677.

[25] Pearson RG. Reasons to Conserve Nature. Trends Ecol Evol. 2016;31(5):366–71. doi: 10.1016/j.tree.2016.02.005 26936225

[26] Smith RD, Maltby E. Using the ecosystem approach to implement the convention on biological diversity: key issues and case studies. IUCN. 2003.

[27] CBD CD. The ecosystem approach (CBD guidelines). Montreal. 2004.

[28] Chan KMA, Shaw MR, Cameron DR, Underwood EC, Daily GC. Conservation planning for ecosystem services. PLoS Biol. 2006;4(11):e379. doi: 10.1371/journal.pbio.0040379 17076586 PMC1629036

[29] Platts PJ, Schaafsma M, Turner RK, Burgess ND, Fisher B, Mbilinyi BP, et al. Inequitable Gains and Losses from Conservation in a Global Biodiversity Hotspot. Environ Resource Econ. 2023;86(3):381–405. doi: 10.1007/s10640-023-00798-y

[30] Msofe NK, Sheng L, Li Z, Lyimo J. Impact of Land Use/Cover Change on Ecosystem Service Values in the Kilombero Valley Floodplain, Southeastern Tanzania. Forests. 2020;11(1):109. doi: 10.3390/f11010109

[31] Mauya EW, Mugasha WA, Njana MA, Zahabu E, Malimbwi R. Carbon stocks for different land cover types in mainland Tanzania. Carbon Balance and Management. 2019;14:1–12. 31030302 10.1186/s13021-019-0120-1 PMC7227115

[32] Sarkar S, Pressey RL, Faith DP, Margules CR, Fuller T, Stoms DM. Biodiversity conservation planning tools: present status and challenges for the future. Annu Rev Environ Resour. 2006;31:123–59.

[33] Marshall E, Wintle BA, Southwell D, Kujala H. What are we measuring? A review of metrics used to describe biodiversity in offsets exchanges. Biological Conservation. 2020;241:108250. doi: 10.1016/j.biocon.2019.108250

[34] Romanelli JP, Meli P, Santos JPB, Jacob IN, Souza LR, Rodrigues AV, et al. Biodiversity responses to restoration across the Brazilian Atlantic Forest. Sci Total Environ. 2022;821:153403. doi: 10.1016/j.scitotenv.2022.153403 35101503

[35] Izsák J, Papp L. A link between ecological diversity indices and measures of biodiversity. Ecological Modelling. 2000;130(1–3):151–6. doi: 10.1016/s0304-3800(00)00203-9

[36] Troudet J, Grandcolas P, Blin A, Vignes-Lebbe R, Legendre F. Taxonomic bias in biodiversity data and societal preferences. Sci Rep. 2017;7(1):9132. doi: 10.1038/s41598-017-09084-6 28831097 PMC5567328

[37] Boakes EH, McGowan PJK, Fuller RA, Chang-qing D, Clark NE, O’Connor K, et al. Distorted views of biodiversity: spatial and temporal bias in species occurrence data. PLoS Biol. 2010;8(6):e1000385. doi: 10.1371/journal.pbio.1000385 20532234 PMC2879389

[38] Townsend Peterson A, Papeş M, Eaton M. Transferability and model evaluation in ecological niche modeling: a comparison of GARP and Maxent. Ecography. 2007;30(4):550–60. doi: 10.1111/j.0906-7590.2007.05102.x

[39] Hughes AC, Grumbine RE. The Kunming-Montreal Global Biodiversity Framework: what it does and does not do, and how to improve it. Front Environ Sci. 2023;11. doi: 10.3389/fenvs.2023.1281536

[40] UNEP-WCMC. Protected Area Profile for United Republic of Tanzania from the World Database on Protected Areas. 2023. https://www.protectedplanet.net/country/TZA

[41] Capitani C, van Soesbergen A, Mukama K, Malugu I, Mbilinyi B, Chamuya N, et al. Scenarios of Land Use and Land Cover Change and Their Multiple Impacts on Natural Capital in Tanzania. Envir Conserv. 2018;46(1):17–24. doi: 10.1017/s0376892918000255

[42] Caro T, Davenport TRB. Wildlife and wildlife management in Tanzania. Conserv Biol. 2016;30(4):716–23. doi: 10.1111/cobi.12658 26681228

[43] Myers N, Mittermeier RA, Mittermeier CG, da Fonseca GA, Kent J. Biodiversity hotspots for conservation priorities. Nature. 2000;403(6772):853–8. doi: 10.1038/35002501 10706275

[44] Brauman KA, Garibaldi LA, Polasky S, Aumeeruddy-Thomas Y, Brancalion PHS, DeClerck F, et al. Global trends in nature’s contributions to people. Proc Natl Acad Sci U S A. 2020;117(51):32799–805. doi: 10.1073/pnas.2010473117 33288690 PMC7768808

[45] MEA. Ecosystems and human well-being. Washington, DC: Island Press. 2005.

[46] Gavin MC, McCarter J, Berkes F, Mead ATP, Sterling EJ, Tang R, et al. Effective Biodiversity Conservation Requires Dynamic, Pluralistic, Partnership-Based Approaches. Sustainability. 2018;10(6):1846. doi: 10.3390/su10061846

[47] Olson DM, Dinerstein E, Wikramanayake ED, Burgess ND, Powell GV, Underwood EC. Terrestrial Ecoregions of the World: A New Map of Life on Earth: A new global map of terrestrial ecoregions provides an innovative tool for conserving biodiversity. BioScience. 2001;51(11):933–8.

[48] Hardy AJ, Gamarra JGP, Cross DE, Macklin MG, Smith MW, Kihonda J, et al. Habitat hydrology and geomorphology control the distribution of malaria vector larvae in rural Africa. PLoS One. 2013;8(12):e81931. doi: 10.1371/journal.pone.0081931 24312606 PMC3849348

[49] TMA TMA. Statement on the status of Tanzania climate in 2019. Dar es Salaam, Tanzania: Tanzania Meteorological Authority. 2019.

[50] Burgess N, Hales JD, Underwood E, Dinerstein E, Olson D, Itoua I. Terrestrial ecoregions of Africa and Madagascar: A conservation assessment. Washington, DC: Island Press. 2004.

[51] UNEP. Ecosystem-Based Adaptation for Rural Resilience in Tanzania. 2016. https://www.thegef.org/projects-operations/projects/5695

[52] Dudley N. Guidelines for applying protected area management categories. IUCN. 2008.

[53] Searle CE, Strampelli P, Smit JB, Mkuburo L, Mathews F, Kiwango H, et al. Spotted hyaena population density across habitat and land use types in southern Tanzania. Journal of Zoology. 2023;322(1):89–100. doi: 10.1111/jzo.13119

[54] Corrigan C, Bingham H, Shi Y, Lewis E, Chauvenet A, Kingston N. Quantifying the contribution to biodiversity conservation of protected areas governed by indigenous peoples and local communities. Biological Conservation. 2018;227:403–12. doi: 10.1016/j.biocon.2018.09.007

[55] Kothari A, Corrigan C, Jonas H, Neumann A, Shrumm H. Recognising and supporting territories and areas conserved by indigenous peoples and local communities: global overview and national case studies. Secretariat of the Convention on Biological Diversity. 2014.

[56] Mittermeier RA, Turner WR, Larsen FW, Brooks TM, Gascon C. Global biodiversity conservation: the critical role of hotspots. Biodiversity hotspots: distribution and protection of conservation priority areas. Springer. 2011:3–22.

[57] Reid AJ, Carlson AK, Creed IF, Eliason EJ, Gell PA, Johnson PTJ, et al. Emerging threats and persistent conservation challenges for freshwater biodiversity. Biol Rev Camb Philos Soc. 2019;94(3):849–73. doi: 10.1111/brv.12480 30467930

[58] Lee TM, Jetz W. Future battlegrounds for conservation under global change. Proc Biol Sci. 2008;275(1640):1261–70. doi: 10.1098/rspb.2007.1732 18302999 PMC2602673

[59] Pillay R, Venter M, Aragon-Osejo J, González-Del-Pliego P, Hansen AJ, Watson JE, et al. Tropical forests are home to over half of the world’s vertebrate species. Front Ecol Environ. 2022;20(1):10–5. doi: 10.1002/fee.2420 35873358 PMC9293027

[60] Buschke FT, Capitani C, Sow EH, Khaemba Y, Kaplin BA, Skowno A, et al. Make global biodiversity information useful to national decision-makers. Nat Ecol Evol. 2023;7(12):1953–6. doi: 10.1038/s41559-023-02226-2 37803167

[61] Salvio GMM, Gomes CR. Protected Area Systems in South American Countries. Floresta Ambient. 2018;25(4). doi: 10.1590/2179-8087.113417

[62] McDonald RI, Boucher TM. Global development and the future of the protected area strategy. Biological Conservation. 2011;144(1):383–92. doi: 10.1016/j.biocon.2010.09.016

[63] Gizachew B, Rizzi J, Shirima DD, Zahabu E. Deforestation and Connectivity among Protected Areas of Tanzania. Forests. 2020;11(2):170. doi: 10.3390/f11020170

[64] Tyrrell P, du Toit JT, Macdonald DW. Conservation beyond protected areas: Using vertebrate species ranges and biodiversity importance scores to inform policy for an east African country in transition. Conservat Sci and Prac. 2019;2(1). doi: 10.1111/csp2.136

[65] Morrison TA, Bolger DT. Connectivity and bottlenecks in a migratory wildebeestConnochaetes taurinuspopulation. Oryx. 2014;48(4):613–21. doi: 10.1017/s0030605313000537

[66] Reiner F, Brandt M, Tong X, Skole D, Kariryaa A, Ciais P, et al. More than one quarter of Africa’s tree cover is found outside areas previously classified as forest. Nat Commun. 2023;14(1):2258. doi: 10.1038/s41467-023-37880-4 37130845 PMC10154416

[67] Coetzee BWT, Gaston KJ, Chown SL. Local scale comparisons of biodiversity as a test for global protected area ecological performance: a meta-analysis. PLoS One. 2014;9(8):e105824. doi: 10.1371/journal.pone.0105824 25162620 PMC4146549

[68] Nana ED, Njabo KY, Tarla FN, Tah EK, Mavakala K, Iponga DM, et al. Putting conservation efforts in Central Africa on the right track for interventions that last. Conservation Letters. 2022;15(6). doi: 10.1111/conl.12913

[69] Hilty J, Worboys GL, Keeley A, Woodley S, Lausche B, Locke H. Guidelines for conserving connectivity through ecological networks and corridors. Best practice protected area Guidelines Series. 2020.

[70] Burgess ND, Butynski TM, Cordeiro NJ, Doggart NH, Fjeldså J, Howell KM, et al. The biological importance of the Eastern Arc Mountains of Tanzania and Kenya. Biological Conservation. 2007;134(2):209–31. doi: 10.1016/j.biocon.2006.08.015

[71] Chowdhury S, Jennions MD, Zalucki MP, Maron M, Watson JEM, Fuller RA. Protected areas and the future of insect conservation. Trends Ecol Evol. 2023;38(1):85–95. doi: 10.1016/j.tree.2022.09.004 36208964

[72] Casanelles-Abella J, Fontana S, Meier E, Moretti M, Fournier B. Spatial mismatch between wild bee diversity hotspots and protected areas. Conserv Biol. 2023;37(4):e14082. doi: 10.1111/cobi.14082 36811162

[73] Wu H, Yu L, Shen X, Hua F, Ma K. Maximizing the potential of protected areas for biodiversity conservation, climate refuge and carbon storage in the face of climate change: A case study of Southwest China. Biological Conservation. 2023;284:110213. doi: 10.1016/j.biocon.2023.110213

[74] Mora C, Sale P. Ongoing global biodiversity loss and the need to move beyond protected areas: a review of the technical and practical shortcomings of protected areas on land and sea. Mar Ecol Prog Ser. 2011;434:251–66. doi: 10.3354/meps09214

[75] Riggio J, Jacobson AP, Hijmans RJ, Caro T. How effective are the protected areas of East Africa?. Global Ecology and Conservation. 2019;17:e00573. doi: 10.1016/j.gecco.2019.e00573

[76] Mozelewski TG, Robbins ZJ, Scheller RM. Forecasting the influence of conservation strategies on landscape connectivity. Conserv Biol. 2022;36(5):e13904. doi: 10.1111/cobi.13904 35212035

[77] Hannah L, Midgley G, Andelman S, Araújo M, Hughes G, Martinez-Meyer E, et al. Protected area needs in a changing climate. Frontiers in Ecology and the Environment. 2007;5(3):131–8. doi: 10.1890/1540-9295(2007)5[131:paniac]2.0.co;2

[78] Williams DR, Rondinini C, Tilman D. Global protected areas seem insufficient to safeguard half of the world’s mammals from human-induced extinction. Proc Natl Acad Sci U S A. 2022;119(24):e2200118119. doi: 10.1073/pnas.2200118119 35666869 PMC9214487

[79] Jones T, Epps C, Coppolillo P, Mbano B, Mutayoba B, Rovero F. Maintaining ecological connectivity between the protected areas of south-central Tanzania: evidence and challenges. Cambridge, UK: Anglia Ruskin University. 2009.

[80] Newmark WD. Isolation of African protected areas. Frontiers in Ecology and the Environment. 2008;6(6):321–8. doi: 10.1890/070003

[81] MNRT. Tanzania wildlife corridor assessment, prioritization, and action plan. 2022.

[82] Grimshaw J, Foley C. Kilimanjaro elephant project 1990: final report. Nairobi: Friends of Conservation. 1990.

[83] Kikoti A, Griffin C, Pamphil L. Elephant use and conflict leads to Tanzania’s first wildlife conservation corridor. Pachyderm. 2010;48:57–66. doi: 10.69649/pachyderm.v48i.234

[84] Tucker MA, Böhning-Gaese K, Fagan WF, Fryxell JM, Van Moorter B, Alberts SC, et al. Moving in the Anthropocene: Global reductions in terrestrial mammalian movements. Science. 2018;359(6374):466–9. doi: 10.1126/science.aam9712 29371471

[85] Joly K, Gurarie E, Sorum MS, Kaczensky P, Cameron MD, Jakes AF, et al. Longest terrestrial migrations and movements around the world. Sci Rep. 2019;9(1):15333. doi: 10.1038/s41598-019-51884-5 31654045 PMC6814704

[86] Gardner TA, Caro T, Fitzherbert EB, Banda T, Lalbhai P. Conservation value of multiple-use areas in East Africa. Conserv Biol. 2007;21(6):1516–25. doi: 10.1111/j.1523-1739.2007.00794.x 18173475

[87] Caro T. Conservation in the African anthropocene. 2015.

[88] Hall C, Dawson TP, Macdiarmid JI, Matthews RB, Smith P. The impact of population growth and climate change on food security in Africa: looking ahead to 2050. International Journal of Agricultural Sustainability. 2017;15(2):124–35. doi: 10.1080/14735903.2017.1293929

[89] Maja MM, Ayano SF. The Impact of Population Growth on Natural Resources and Farmers’ Capacity to Adapt to Climate Change in Low-Income Countries. Earth Syst Environ. 2021;5(2):271–83. doi: 10.1007/s41748-021-00209-6

[90] Caro TM. Factors Affecting the Small Mammal Community Inside and Outside Katavi National Park, Tanzania1. Biotrop. 2002;34(2):310. doi: 10.1646/0006-3606(2002)034[0310:fatsmc]2.0.co;2

[91] Hezron E, Ngondya IB, Munishi LK. Roles of Maasai Alalili Systems in Sustainable Conservation of Fodder Species of East African Rangelands. Rangeland Ecology & Management. 2025;98:490–507. doi: 10.1016/j.rama.2024.10.007

[92] Tarimo F, Kelly C, Moyo F, Munishi L. Importance of Traditional Ecological Knowledge in Promoting Biodiversity Conservation Outside Protected Areas. Hum Ecol.

[93] Hilty J, Worboys GL, Keeley A, Woodley S, Lausche BJ, Locke H. Guidelines for conserving connectivity through ecological networks and corridors. 2020.

[94] Harvey E, Gounand I, Ward CL, Altermatt F. Bridging ecology and conservation: from ecological networks to ecosystem function. Journal of Applied Ecology. 2016;54(2):371–9. doi: 10.1111/1365-2664.12769

[95] Liczner AR, Pither R, Bennett JR, Bowman J, Hall KR, Fletcher RJ Jr, et al. Advances and challenges in ecological connectivity science. Ecol Evol. 2024;14(9):e70231. doi: 10.1002/ece3.70231 39224156 PMC11366504

[96] König C, Weigelt P, Schrader J, Taylor A, Kattge J, Kreft H. Biodiversity data integration-the significance of data resolution and domain. PLoS Biol. 2019;17(3):e3000183. doi: 10.1371/journal.pbio.3000183 30883539 PMC6445469

[97] Urban MC, Bocedi G, Hendry AP, Mihoub J-B, Pe’er G, Singer A, et al. Improving the forecast for biodiversity under climate change. Science. 2016;353(6304):aad8466. doi: 10.1126/science.aad8466 27609898

