# Supplementary material for: Using biodiversity features to promote an Ecosystem-Based Conservation framework in terrestrial ecosystems
Source: PLoS One. 2025 Nov 21;20(11):e0336705. doi: 10.1371/journal.pone.0336705 (PMC12637941; doi:10.1371/journal.pone.0336705)
Supplement: S1 Table — This table presents search terms, databases (Google Scholar and Scopus), total articles retrieved, relevant articles identified, and those included for data extraction for vertebrates, invertebrates, and plants across each ecosystem type. (PDF) [file pone.0336705.s001.pdf]

S1 Table: Summary of literature search results for taxa-specific biodiversity studies across eight terrestrial ecosystems in Tanzania.

Ecosystem type	*Search terms used*	*Total articles retrieved*	*Relevant articles identified*	*Articles included for data extraction*
Acacia savanna	-Vertebrates; [(Acacia savanna* OR Acacia savanna AND habitat* OR Acacia savanna AND ecosystem*) AND (biodiversity OR diversity) AND (Tanzania*) AND (vertebrate* OR mammal* OR reptile* OR amphibian* OR herpetofauna* OR bird)]	GS=2740	51	5
		Scopus=568	49	
	-Invertebrates; [(Acacia savanna* OR Acacia savanna AND habitat* OR Acacia savanna AND ecosystem*) AND (biodiversity OR diversity) AND (Tanzania*) AND (invertebrate* OR insect* OR mollusc* OR crustacean* OR annelid* OR arachnid* OR macroinvertebrate*)]	GS=2010	44	9
		Scopus=256	18	
	-Plants; [(Acacia savanna* OR Acacia savanna AND habitat* OR Acacia savanna AND ecosystem*) AND (biodiversity OR diversity) AND (Tanzania*) AND (flora* OR plant* OR woody AND plant* OR tree* OR shrub OR grass* OR herb* OR lower AND plant*)]	GS=8430	60	5
		Scopus=371	42	
Flooded savanna	-Vertebrates; [(Flooded savanna* OR Flooded savanna AND habitat* OR Flooded savanna AND ecosystem*) AND (biodiversity OR diversity) AND (Tanzania*) AND (vertebrate* OR mammal* OR reptile* OR amphibian* OR herpetofauna* OR bird)]	GS=3890	39	2
		Scopus=192	5	
	-Invertebrates; [(Flooded savanna* OR Flooded savanna AND habitat* OR Flooded savanna AND ecosystem*) AND (biodiversity OR diversity) AND (Tanzania*) AND (invertebrate* OR insect* OR mollusc* OR crustacean* OR annelid* OR arachnid* OR macroinvertebrate*)]	GS=3010	24	0
		Scopus=88	4	

	-Plants; [(Flooded savanna* OR Flooded savanna AND habitat* OR Flooded savanna AND ecosystem*) AND (biodiversity OR diversity) AND (Tanzania*) AND (flora* OR plant* OR woody AND plant* OR tree* OR shrub OR grass* OR herb* OR lower AND plant*)]	GS=11638	13	2
		Scopus=805	10	
Coastal forest	-Vertebrates; [(Coastal forest* OR Coastal forest AND habitat* OR Coastal forest AND ecosystem*) AND (biodiversity OR diversity) AND (Tanzania*) AND (vertebrate* OR mammal* OR reptile* OR amphibian* OR herpetofauna* OR bird)]	GS= 12,700	84	15
		Scopus=570	169	
	-Invertebrates; [(Coastal forest* OR Coastal forest AND habitat* OR Coastal forest AND ecosystem*) AND (biodiversity OR diversity) AND (Tanzania*) AND (invertebrate* OR insect* OR mollusc* OR crustacean* OR annelid* OR arachnid* OR macroinvertebrate*)]	GS=12,200	52	2
		Scopus=1761	115	
	-Plants; [(Coastal forest* OR Coastal forest AND habitat* OR Coastal forest AND ecosystem*) AND (biodiversity OR diversity) AND (Tanzania*) AND (flora* OR plant* OR woody AND plant* OR tree* OR shrub OR grass* OR herb* OR lower AND plant*)]	GS=23,500	149	15
		Scopus = 4,012	203	
Montane forest	-Vertebrates; [(Montane forest* OR Montane forest AND habitat* OR Montane forest AND ecosystem*) AND (biodiversity OR diversity) AND (Tanzania*) AND (vertebrate* OR mammal* OR reptile* OR amphibian* OR herpetofauna* OR bird)]	GS=6,100	86	46
		Scopus=1515	131	
	-Invertebrates; [(Montane forest* OR Montane forest AND habitat* OR Montane forest AND ecosystem*) AND (biodiversity OR diversity) AND (Tanzania*) AND (invertebrate* OR insect* OR mollusc* OR crustacean* OR annelid* OR arachnid* OR macroinvertebrate*)]	GS= 8196	40	17
		Scopus=609	54	
	-Plants; [(Montane forest* OR Montane forest AND habitat* OR Montane forest AND ecosystem*) AND (biodiversity OR diversity) AND (Tanzania*) AND (flora* OR plant* OR woody AND plant* OR tree* OR shrub OR grass* OR herb* OR lower AND plant*)]	GS=14,200	134	33
		Scopus=304	143	
Mangrove		GS= 1610	30	3

	-Vertebrates; [(Mangrove* OR Mangrove AND habitat* OR Mangrove AND ecosystem*) AND (biodiversity OR diversity) AND (Tanzania*) AND (vertebrate* OR mammal* OR reptile* OR amphibian* OR herpetofauna* OR bird)]	Scopus=296	17	
	-Invertebrates; [(Mangrove* OR Mangrove AND habitat* OR Mangrove AND ecosystem*) AND (biodiversity OR diversity) AND (Tanzania*) AND (invertebrate* OR insect* OR mollusc* OR crustacean* OR annelid* OR arachnid* OR macroinvertebrate*)]	GS=2250	54	8
		Scopus=762	104	
	-Plants; [(Mangrove* OR Mangrove AND habitat* OR Mangrove AND ecosystem*) AND (biodiversity OR diversity) AND (Tanzania*) AND (flora* OR plant* OR woody AND plant* OR tree* OR shrub OR grass* OR herb* OR lower AND plant*)]	GS=6980	168	7
		Scopus=590	44	
Miombo woodland	-Vertebrates; [(Miombo woodland* OR Miombo woodland AND habitat* OR Miombo woodland AND ecosystem*) AND (biodiversity OR diversity) AND (Tanzania*) AND (vertebrate* OR mammal* OR reptile* OR amphibian* OR herpetofauna* OR bird)]	GS= 1240	53	18
		Scopus=1597	107	
	-Invertebrates; [(Miombo woodland* OR Miombo woodland AND habitat* OR Miombo woodland AND ecosystem*) AND (biodiversity OR diversity) AND (Tanzania*) AND (invertebrate* OR insect* OR mollusc* OR crustacean* OR annelid* OR arachnid* OR macroinvertebrate*)]	GS=902	33	4
		Scopus=1237	23	
	Plants; [(Miombo woodland* OR Miombo woodland AND habitat* OR Miombo woodland AND ecosystem*) AND (biodiversity OR diversity) AND (Tanzania*) AND (flora* OR plant* OR woody AND plant* OR tree* OR shrub OR grass* OR herb* OR lower AND plant*)]	GS=4590	137	12
		Scopus=1139	213	
Moorland	-Vertebrates; [(Moorland* OR Moorland AND habitat* OR Moorland AND ecosystem*) AND (biodiversity OR diversity) AND (Tanzania*) AND (vertebrate* OR mammal* OR reptile* OR amphibian* OR herpetofauna* OR bird)]	GS=1143	14	2
		Scopus=63	3	
		GS=1000	0	0

	-Invertebrates; [(Moorland* OR Moorland AND habitat* OR Moorland AND ecosystem*) AND (biodiversity OR diversity) AND (Tanzania*) AND (invertebrate* OR insect* OR mollusc* OR crustacean* OR annelid* OR arachnid* OR macroinvertebrate*)]	Scopus=85	0	
	-Plants; [(Moorland* OR Moorland AND habitat* OR Moorland AND ecosystem*) AND (biodiversity OR diversity) AND (Tanzania*) AND (flora* OR plant* OR woody AND plant* OR tree* OR shrub OR grass* OR herb* OR lower AND plant*)]	GS=2650	22	1
		Scopus= 174	3	
Grassland	-Vertebrates; [(Grassland* OR Grassland AND habitat* OR Grassland AND ecosystem*) AND (biodiversity OR diversity) AND (Tanzania*) AND (vertebrate* OR mammal* OR reptile* OR amphibian* OR herpetofauna* OR bird)]	GS= 9580	76	11
		Scopus=1587	234	
	-Invertebrates; [(Grassland* OR Grassland AND habitat* OR Grassland AND ecosystem*) AND (biodiversity OR diversity) AND (Tanzania*) AND (invertebrate* OR insect* OR mollusc* OR crustacean* OR annelid* OR arachnid* OR macroinvertebrate*)]	GS=7574	59	4
		Scopus=1615	120	
	-Plants; [(Grassland* OR Grassland AND habitat* OR Grassland AND ecosystem*) AND (biodiversity OR diversity) AND (Tanzania*) AND (flora* OR plant* OR woody AND plant* OR tree* OR shrub OR grass* OR herb* OR lower AND plant*)]	GS=19700	37	7
		Scopus= 4685	247	

GS = Google Scholor search engine
