# Supplementary material for: Using biodiversity features to promote an Ecosystem-Based Conservation framework in terrestrial ecosystems
Source: PLoS One. 2025 Nov 21;20(11):e0336705. doi: 10.1371/journal.pone.0336705 (PMC12637941; doi:10.1371/journal.pone.0336705)
Supplement: S2 Data — The dataset summarises the sum of taxonomic group (vertebrates, invertebrates, plants) species counts, and their corresponding references. (PDF) [file pone.0336705.s002.pdf]

Ecosystem Type	No. of Species Reported	Taxa Reported	Reporting Articles	Study Site
Mangrove	47	Vertebrates	(Howell et al., 2000)	Rufiji floodplain and delta
	2	Vertebrates	(Mangora et al., 2016)	Mangroves of the Western Indian Ocean
	15	Vertebrates	(Semesi, 1992)	Mangrove of Tanzania
	7	Invertebrates	(Wagner et al., 2004)	Mangrove Forests in Mnazi Bay
	1	Invertebrates	(Rumisha et al., 2018)	Mangroves of mainland Tanzania
	13	Invertebrates	(Mangora et al., 2016)	Mangroves of the Western Indian Ocean
	1	Invertebrates	(Nehemia & Kochzius, 2019)	Mangroves of Tanzania
	2	Invertebrates	(Nehemia, Ng endu, et al., 2019)	Mangrove, Tanzania
	13	Invertebrates	(Rumisha et al., 2015)	Mangroves, Tanzania
	4	Invertebrates	(Torres et al., 2008)	Mangroves at Mngoji site
	11	Invertebrates	(Semesi, 1992)	Mangroves of Tanzania
	18	Plants	(Mangora et al., 2016)	Mangroves of the Western Indian Ocean
	6	Plants	(Semesi, 1992)	Mangroves of Tanzania
	8	Plants	(Njana, 2020)	Mangrove forest
	9	Plants	(Njana et al., 2018)	Mangrove forest
	9	Plants	(Mungai et al., 2019)	Transboundary area of Kenya and Tanzania
	5	Plants	(Wagner et al., 2004)	Mangrove Forests in Mnazi Bay Ruvuma Estuary

	6	Plants	(Alavaisha & Mangora, 2016)	small Estuarine Mangroves of Geza and Mtimbwani
Coastal Forest	154	Vertebrates	(Gwegime et al., 2014)	Rondo nature reserve
	10	Vertebrates	(Sabuni et al., 2015)	Tanzanian coastal forests
	161	Vertebrates	(Ansell & Dickinson, 1994)	Zaraninge Kiono
	51	Vertebrates	(Barratt, 2017)	Coastal forest of Tanzania
	65	Vertebrates	(Burgess et al., 2017)	Coastal forest of Tanzania
	1	Vertebrates	(Barratt et al., 2017)	Coastal forest of Tanzania
	466	Vertebrates	(Clarke, 1995)	Coastal forests of Lindi
	560	Vertebrates	(Clarke & Stubblefield, 1995)	Coastal forest of Tanga
	11	Vertebrates	(Kiwia, 2006)	Zaraninge Forest
	93	Vertebrates	(Mbije & Kamungu, 2021)	Kimboza Forest reserve
	57	Vertebrates	(Modest & Hassan, 2016)	Coastal forest of Tanzania
	268	Vertebrates	(Rija et al., 2015)	Saadani NP
	2	Vertebrates	(Sabuni et al., 2016)	Coastal forest
	205	Vertebrates	(Tanzania et al., 2012)	Namatibili gorge, Mitundumbea and Mapatwa forests
	72	Vertebrates	(Werema, 2021)	Pangani
	56	Invertebrates	(Masikini et al., 2018)	Along the Zigi River – Tanzania
	133	Invertebrates	(Mohamed, 2023)	Dar es salaam urban remnant forest
	87	Plants	(Ntukey et al., 2022)	Coastal forest

	285	Plants	(Ansell & Dickinson, 1994)	Zaraninge Kiono
	63	Plants	(Kimaro, 2007)	Ngumburuni Forest Reserve
	312	Plants	(Mligo, 2015)	Namatimbili Forest
	375	Plants	(Tanzania et al., 2012)	Coastal forest
	261	Plants	(Mligo, 2017)	The Wami River system
	116	Plants	(Mligo, 2019)	Pugu Forest reserve
	76	Plants	(Mligo et al., 2009)	Zaraninge Forest
	150	Plants	(Lovett et al., 2001)	Coastal forests
	146	Plants	(Mwasumbi et al., 1994)	Pande and Kiono coastal forests
	110	Plants	(Mligo, 2018)	Ruvu South Forest Reserve
	449	Plants	(Doody & Hamerlynck, 2003)	Rufiji district
	377	Plants	(Mwasumbi et al., 2000)	Forests in the Rufiji floodplain
	131	Plants	(Washa, 2023)	Mitarure Forest Reserve
	297	Plants	(Gwegime et al., 2014)	Rondo Nature reserve
Miombo woodland	19	Vertebrates	(D'Ammando et al., 2022)	Issa valley Tanzania
	10	Vertebrates	(Jenkins et al., 2003)	Teak plantation - Kilombero valley
	156	Vertebrates	(Werema & Nahonyo, 2022)	Miombo woodlands in Mba-rang'andu and Kimbanda WMA
	23	Vertebrates	(Fitzherbert et al., 2007)	In and around Katavi NP
	24	Vertebrates	(Caro, 1999a)	Katavi NP
	12	Vertebrates	(Caro, 2002)	In and around Katavi

	84	Vertebrates	(Caro et al., 2011)	Katavi National Park
	19	Vertebrates	(Waltert et al., 2008)	Western Tanzania
	24	Vertebrates	(Caro, 1999b)	NP, GCA and open area in Katavi ecosystem
	148	Vertebrates	(John & Kiwango, 2021)	Isunkaviola, western part of Ruaha National Park
	8	Vertebrates	(Saanya et al., 2023)	Selous Game Reserve
	36	Vertebrates	(Piel et al., 2019)	Issa valley
	22	Vertebrates	(Mtui et al., 2017)	Ruaha Rungwa Katavi ecosystem
	10	Vertebrates	(Butynski & de Jong, 2009)	Mahale NP
	458	Vertebrates	(Engilis Jr et al., 2009)	Katavi Rukwa ecosystem
	28	Vertebrates	(Gardner et al., 2007)	Miombo- mopane woodlands, western Tanzania.
	3	Vertebrates	(Halima & Fraser, 2022)	Usangu area
	85	Vertebrates	(Mgelwa et al., 2023)	Around Lake Rukwa
	142	Invertebrates	(Hemp & Heller, 2019)	Miombo woodlands
	104	Invertebrates	(Jew et al., 2015)	Miombo woodland in South-West Tanzania
	186	Invertebrates	(Fitzherbert et al., 2006)	In and around Katavi National Park
	172	Invertebrates	(Giliba, 2023)	Katavi-Rukwa Ecosystem
	48	Plants	(Maliondo et al., 2005)	Madebe, one of the villages in Handeni district
	86	Plants	(Backéus et al., 2006)	Miombo woodland area, Estern Tanzania

	229	Plants	(Banda et al., 2008)	Dry forest miombo woodland in the Katavi-Rukwa ecosystem
	118	Plants	(Ruvuga et al., 2021)	Miombo woodland of Eastern Tanzania
	217	Plants	(Shirima et al., 2015)	Wet and dry miombo woodlands of Tanzania.
	110	Plants	(Giliba et al., 2011)	Miombo woodland of Bereku reserve
	17	Plants	(Seki et al., 2018)	Miombo woodland in South eastern and western Tanzania
	3	Plants	(Darbyshire & Ndangalasi, 2008)	Western Tanzania
	13	Plants	(Nyomora, 2005)	Iringa, Mbeya, Rukwa and Ruvuma
	57	Plants	(John, 2018)	Mkulazi Catchment Forest Reserve
	71	Plants	(Lyimo & Shaaban, 2015)	Kitulangal Forest reserve
	39	Plants	(Munishi et al., 2011)	Near lake Rukwa basin Chunya District
Montane Forest	50	Vertebrates	(Mapunda & John, 2021)	Magamba nature forest reserve
	21	Vertebrates	(Cordeiro et al., 2006)	Udagaje and kihansi gorges
	43	Vertebrates	(Rossi et al., 2010)	Bomalangome and mapanda sites Udzungwa
	45	Vertebrates	(Lyakurwa et al., 2019)	Uzungwa scarp nature reserve
	10	Vertebrates	(Kisingo et al., 2005)	The Lulanda forest in Udzungwa
	28	Vertebrates	(C. Werema, 2015)	Mangala Forest in slopes of Uluguru
	129	Vertebrates	(Rovero, Menegon, et al., 2014)	Eastern arc Mts of Tanzania
	16	Vertebrates	(Seddon et al., 1999)	Nilo and Nguu North Forest
	10	Vertebrates	(Stanley & Kihaule, 2016)	Mt. Meru mountain

	49	Vertebrates	(Cordeiro et al., 2015)	East Usambara Mountains
	161	Vertebrates	(Doggart et al., 2006)	Rubeho Mountains
	14	Vertebrates	(Liedtke et al., 2022)	Ukaguru mountains
	15	Vertebrates	(Gebrezgiher et al., 2022)	Mount Meru
	14	Vertebrates	(Thomas et al., 2022)	West Mount Kilimanjaro
	20	Vertebrates	(Mulungu et al., 2008)	Mount Kilimanjaro
	6	Vertebrates	(Menegon et al., 2022)	Eastern arc Mts of Tanzania
	185	Vertebrates	(Byamungu et al., 2021)	Southern slopes of Mt. Kilimanjaro,
	8	Vertebrates	(Richard et al., 2022)	Mount Rungwe Nature Forest Reserve
	9	Vertebrates	(Rovero et al., 2009)	Udzungwa Mountains
	26	Vertebrates	(Rovero, Martin, et al., 2014)	Udzungwa Mountains
	1	Vertebrates	(Lawson et al., 2023)	Ukaguru mountains
	23	Vertebrates	(Katunzi et al., 2021)	Chome Nature Forest reserve
	6	Vertebrates	(Chidodo et al., 2020)	North Uluguru Mts
	65	Vertebrates	(Menegon et al., 2011)	Mahenge Mountains Forest
	1	Vertebrates	(Rovero et al., 2008)	Udzungwa Mountains
	13	Vertebrates	(Ademola et al., 2022)	Ukaguru Mountains
	7	Vertebrates	(Lema & Magige, 2018)	Agricultural land near Kindroko forest
	5	Vertebrates	(Lema & Magige, 2018)	Kindoroko forest reserve in North Pare
	308	Vertebrates	(Fjeldså et al., 2010)	Eastern arc Mountains
	92	Vertebrates	(Doggart et al., 2008)	North Pare Mountains

	11	Vertebrates	(Stanley et al., 2005)	Kwamgumi Forest Reserve, East Usambara Mountains
	26	Vertebrates	(Werema & Howell, 2016)	Bunduki Forest Reserve in the Uluguru Mountains
	27	Vertebrates	(Jones et al., 2019)	Udzungwa Mountains
	54	Vertebrates	(Mkonyi, 2021)	Uluguru Mountain Forest Reserves,
	46	Vertebrates	(Stanley et al., 2011)	East and West Usambara Mountains
	16	Vertebrates	(Trentin & Rovero, 2011)	Uzungwa Scarp Forest Reserve
	11	Vertebrates	(Lawson & Moyer, 2008)	Udzungwa Plateau
	28	Vertebrates	(Vihemäki et al., 2013)	East Usambara
	28	Vertebrates	(Chacha Werema, 2015)	Mangala Forest Reserve
	10	Vertebrates	(Sabuni et al., 2018)	Mt. Kitumbeine, northern highland
	16	Vertebrates	(Stanley, 2015)	Kilimanjaro and Meru mountains
	118	Vertebrates	(Jones, 2013)	Udzungwa Mountains
	64	Vertebrates	(Werema et al., 2012)	Ikokoto Forest, Udzungwa Mountains
	5	Vertebrates	(Stanley et al., 2007)	Kindoroko and Minja Forest Reserves, North Pare
	1	Vertebrates	(Menegon et al., 2002)	Udzungwa Mountains of Tanzania,
	33	Vertebrates	(Gebert, 2022)	Mount Kilimanjaro
	279	Invertebrates	(Axmacher et al., 2009)	South-western slopes of Mount Kilimanjaro
	18	Invertebrates	(Liseki & Vane-Wright, 2011)	Mount Kilimanjaro

	72	Invertebrates	(Rija, 2022)	Kihansi gorge ecosystem in Southern Udzungwa Mountains,
	101	Invertebrates	(Kunene et al., 2022)	Udzungwa Mountains
	302	Invertebrates	(Axmacher & Fiedler, 2008)	Mt Kilimanjaro, Tanzania
	149	Invertebrates	(Sørensen, 2004)	Uzungwa Mountains of Tanzania
	33	Invertebrates	(Zilihona & Nummelin, 2001)	Near Kihansi waterfall, in the Udzungwa Mountains
	32	Invertebrates	(Zilihona et al., 2004)	Udzungwa Mountains
	28	Invertebrates	(Robertson, 2002)	Montane forest of Mkomazi, south pare and West usambara
	188	Invertebrates	(Axmacher, Holtmann, et al., 2004)	South-western slopes of Mt. Kilimanjaro,
	170	Invertebrates	(Sørensen et al., 2002)	Uzungwa Scarp Forest Reserve
	101	Invertebrates	(Kunene, 2020)	Udzungwa Mountains
	16	Invertebrates	(Geeraert, 2014)	Amani Nature Reserve
	28	Invertebrates	(Sørensen, 1993)	Uluguru mountains
	19	Invertebrates	(Notø, 2014)	Amani Nature Reserve
	29	Invertebrates	(Hemp, 2013)	East Usambara Mts
	35	Invertebrates	(Sæbjørnsen, 2016)	Amani Nature Reserve
	115	Plants	(Kikoti & Mligo, 2015)	Montane forests on the northern slope of Mount Kilimanjaro.

	962	Plants	(Mollel et al., 2017)	Mount Kilimanjaro along elevation gradient
	70	Plants	(Mapunda & John, 2021)	Magamba nature forest reserve
	132	Plants	(Kikoti et al., 2022)	Lower montane Forest of Kilimanjaro
	79	Plants	(Mwaluseke et al., 2023)	Dry evergreen montane fo the rest of Lendikinya forest reserve
	60	Plants	(Mwendwa et al., 2020)	Amani Nature Forest Reserve
	447	Plants	(Axmacher et al., 2009)	south-western slopes of Mount Kilimanjaro
	35	Plants	(Lasway et al., 2023)	Mt. Meru forest
	194	Plants	(Hall et al., 2011)	Upland East Usambara
	93	Plants	(Axmacher, Tünte, et al., 2004)	south-western slopes of Mt Kilimanjaro
	140	Plants	(Hemp, 2002)	Southern slopes of Mt. Kilimanjaro.
	1220	Plants	(Hemp, 2006)	Forests of Mt. Kilimanjaro
	108	Plants	(Temu & Andrew, 2008)	Uluguru Mountains
	100	Plants	(Msuya & Kideghesho, 2009)	West Usambara Mountains
	104	Plants	(Lyaruu et al., 2000)	dry Afromontane Forest at Mafai
	262	Plants	(Munishi et al., 2004)	Usambara mountains
	445	Plants	(Munishi et al., 2004)	Uluguru mts
	102	Plants	(Mattila & Koponen, 1999)	Mt. Meru and Mt Usambara
	110	Plants	(Renner et al., 2022)	Mt. Kilimanjaro
	204	Plants	(Lovett et al., 2006)	Udzungwa Mountain NP
	240	Plants	(Lovett et al., 2001)	Eastern arc and Northern Forests

	54	Plants	(MWAKALUKWA et al., 2023)	Essimingor Nature Forest Reserve
	489	Plants	(Shangali et al., 1998)	North-western and southern parts of the Uzungwa Scarp Forest Reserve
	308	Plants	(Kayombo, Koka, et al., 2022)	Monduli Mountain Forest Reserve
	162	Plants	(Vihemäki et al., 2013)	East Usambaras
	169	Plants	(Lovett, 1996)	Udzungwa Mountain
	111	Plants	(Lovett, 1996)	West Usambara Mountains
	62	Plants	(Lovett, 1996)	Nguru Mountains
	147	Plants	(Kayombo et al., 2020)	Afromontane Vegetation of Nkoanenkole Forest Reserve (NCFR)
	153	Plants	(Kayombo, Ndangalasi, et al., 2022)	Image Forest reserve in Eeastern arc mountains
	39	Plants	(Sitati et al., 2014)	Gelai Forest Reserve
	55	Plants	(Ojoyi et al., 2015)	Uluguru forest
	82	Plants	(Marshall, 2007)	Magombera forest
	128	Vertebrates	(Werema, 2021)	Serengeti Ecosystem
	45	Vertebrates	(Byrom et al., 2014)	Greater Serengeti Ecosystems
	14	Vertebrates	(Sangiwa & Magige, 2019)	Northern Serengeti
	22	Vertebrates	(Nasseri, 2009)	North east Tanzania
	9	Vertebrates	(Loftis, 2015)	Manyara Ranch
	229	Invertebrates	(Russell-Smith, 2002)	Mkomazi Game reserve

	102	Invertebrates	(de Visser et al., 2015)	Serengeti
	45	Invertebrates	(Stanbrook et al., 2021)	wakuchinja wildlife corridor
	492	Invertebrates	(Dell et al.)	Mkomazi Game reserve
	20	Invertebrates	(Villet et al., 1999)	Mkomazi Game reserve
	4	Invertebrates	(Smith & Jocqué, 2015)	Mkomazi Game reserve
	19	Invertebrates	(Gayo, 2022)	University of Dodoma
	69	Invertebrates	(Wesolowska & Russell-Smith, 2000)	Mkomazi game reserve
	492	Invertebrates	(Krüger & MCGAVIN, 1997)	Mkomazi game reserve
	14	Plants	(Mduma et al., 2007)	Serengeti National Park
	118	Plants	(Levi et al., 2022)	Tarangire Ecosystem
	29	Plants	(Mercader et al., 2019)	Eastern Serengeti Plains,
	6	Plants	(Krüger & MCGAVIN, 1997)	Mkomazi game reserve
	314	Plants	(Mligo, 2015)	Serengeti Ecosystem
Grassland	67	Vertebrates	(Sinclair et al., 2002)	Serengeti NP
	8	Vertebrates	(Magige, 2013)	Northern Serengeti
	6	Vertebrates	(Manyonyi et al., 2020)	Serengeti NP
	11	Vertebrates	(Moehlman et al., 2020)	Ngorongoro Crater
	24	Vertebrates	(Hassan & Rija, 2011)	Serengeti National Park
	9	Vertebrates	(Shilereyo et al., 2021)	Serengeti Ecosystem
	14	Vertebrates	(Kiffner et al., 2020)	Manyara Ranch

	26	Vertebrates	(Durant et al., 2010)	Serengeti -Ngorongoro landscape
	98	Vertebrates	(Nkwabi et al., 2018)	Serengeti NP and Ngorongoro CA
	18	Vertebrates	(Timbuka & Kabigumila, 2006)	Serengeti Kopjes
	84	Vertebrates	(Msindai, 2014)	Serengeti National Park
	239	Invertebrates	(Mpondo et al., 2021)	Simanjiro, Northern Tanzania
	183	Invertebrates	(Lasway et al., 2022)	Areas surrounding Tarangire National Park, the lowlands of Mt. Meru and Mt. Kilimanjaro
	45	Invertebrates	(Lasway et al., 2021)	Northern Tanzania
	45	Invertebrates	(Stanbrook et al., 2021)	KWC
	16	Plants	(Andrews et al., 2011)	Endulen-Laetoli
	30	Plants	(Mseja et al., 2020)	KWC
	123	Plants	(Kavana et al., 2019)	Serengeti
	15	Plants	(Bukombe et al., 2018)	Serengeti National Park
	10	Plants	(Andrews & Bamford, 2008)	Laetoli, Northern Tanzania
	314	Plants	(Mligo, 2015)	Serengeti ecosystem
	14	Plants	(Musese et al., 2020)	Simanjiro Rangeland,
Flooded savanna	67	Vertebrates	(Soka et al., 2013)	Hombolo lake
	6	Vertebrates	(Jenkins et al., 2002)	Kilombero valley
	115	Plants	(Andrew et al., 2015)	Kilombero Valley
	115	Plants	(Andrew et al., 2012)	Kilombero Valley

Moorland	6	Vertebrates	(Thomas et al., 2022)	Moorland of Mount Kilimanjaro
	18	Vertebrates	(Stanley et al., 2014)	Mt. Kilimanjaro
	5	Plants	(Hemp, 2011)	Mount Kilimanjaro

References

1. Ademola, O. J., Massawe, A. W., Mulungu, L. S., Hieronimo, P., Makonda, F. B., & Makundi, R. H. (2022). Habitat type impacts small mammal diversity in the Ukaguru Mountains, Tanzania. Mammalia, 86(2), 123-133.
2. Alavaisha, E., & Mangora, M. M. (2016). Carbon stocks in the small estuarine mangroves of Geza and Mtimbwani, Tanga, Tanzania. International Journal of Forestry Research, 2016.
3. Andrew, S. M., Moe, S. R., Totland, Ø., & Munishi, P. K. (2012). Species composition and functional structure of herbaceous vegetation in a tropical wetland system. Biodiversity and Conservation, 21, 2865-2885.
4. Andrew, S. M., Totland, Ø., & Moe, S. R. (2015). Spatial variation in plant species richness and diversity along human disturbance and environmental gradients in a tropical wetland. Wetlands ecology and management, 23, 395-404.
5. Andrews, P., & Bamford, M. (2008). Past and present vegetation ecology of Laetoli, Tanzania. Journal of Human Evolution, 54(1), 78-98.
6. Andrews, P., Bamford, M. K., Njau, E. F., & Leliyo, G. (2011). The ecology and biogeography of the endulen-laetoli area in northern Tanzania. In Vertebrate Paleobiology and Paleoanthropology (pp. 167-200). Springer. https://doi.org/10.1007/978-90-481-9956-3_8

7. Ansell, C., & Dickinson, A. (1994). Site Description and Conservation Evaluation.
8. Axmacher, J. C., & Fiedler, K. (2008). Habitat type modifies geometry of elevational diversity gradients in geometrid moths (Lepidoptera Geometridae) on Mt Kilimanjaro, Tanzania. Tropical Zoology, 21(2), 243-251.
9. Axmacher, J. C., Brehm, G., Hemp, A., Tünte, H., Lyaruu, H. V. M., Müller-Hohenstein, K., & Fiedler, K. (2009). Determinants of diversity in afrotropical herbivorous insects (Lepidoptera: Geometridae): Plant diversity, vegetation structure or abiotic factors? [Article]. Journal of Biogeography, 36(2), 337-349. https://doi.org/10.1111/j.1365-2699.2008.01997.x
10. Axmacher, J. C., Holtmann, G., Scheuermann, L., Brehm, G., Müller-Hohenstein, K., & Fiedler, K. (2004). Diversity of geometrid moths (Lepidoptera: Geometridae) along an Afrotropical elevational rainforest transect. Diversity and Distributions, 10(4), 293-302.
11. Axmacher, J. C., Tünte, H., Schrumpf, M., Müller-Hohenstein, K., Lyaruu, H. V., & Fiedler, K. (2004). Diverging diversity patterns of vascular plants and geometrid moths during forest regeneration on Mt Kilimanjaro, Tanzania. Journal of Biogeography, 31(6), 895-904.
12. Backéus, I., Pettersson, B., Strömquist, L., & Ruffo, C. (2006). Tree communities and structural dynamics in miombo (Brachystegia-Julbernardia) woodland, Tanzania [Article]. Forest Ecology and Management, 230(1-3), 171-178. https://doi.org/10.1016/j.foreco.2006.04.033
13. Banda, T., Mwangulango, N., Meyer, B., Schwartz, M. W., Mbago, F., Sungula, M., & Caro, T. (2008). The woodland vegetation of the Katavi-Rukwa ecosystem in western Tanzania [Article]. Forest Ecology and Management, 255(8-9), 3382-3395. https://doi.org/10.1016/j.foreco.2008.01.079
14. Barratt, C. D. (2017). Biodiversity patterns and conservation of the coastal forests of Eastern Africa University_of_Basel].
15. Barratt, C. D., Lawson, L. P., Bittencourt-Silva, G. B., Doggart, N., Morgan-Brown, T., Nagel, P., & Loader, S. P. (2017). A new, narrowly distributed, and critically endangered species of spiny-throated reed frog (Anura: Hyperoliidae) from a highly threatened coastal forest reserve in Tanzania. Herpetological Journal, 27(1).

16. Bukombe, J., Smith, S. W., Kija, H., Loishooki, A., Sumay, G., Mwita, M., . . . Kihwele, E. (2018). Fire regulates the abundance of alien plant species around roads and settlements in the Serengeti National Park [Article]. Management of Biological Invasions, 9(3), 357-367. https://doi.org/10.3391/mbi.2018.9.3.17

17. Burgess, N. D., Malugu, I., Sumbi, P., Kashindye, A., Kijazi, A., Tabor, K., . . . Gereau, R. E. (2017). Two decades of change in state, pressure and conservation responses in the coastal forest biodiversity hotspot of Tanzania. Oryx, 51(1), 77-86.

18. Butynski, T. M., & de Jong, Y. A. (2009). Primates of Mahale Mountains National Park, Tanzania. Greystoke Mahale Camp. Website:< http://www. wildsolutions. nl.

19. Byamungu, R. M., Schleuning, M., Ferger, S. W., Helbig-Bonitz, M., Hemp, A., Neu, A., . . . Albrecht, J. (2021). Abiotic and biotic drivers of functional diversity and functional composition of bird and bat assemblages along a tropical elevation gradient [Article]. Diversity and Distributions, 27(12), 2344-2356. https://doi.org/10.1111/ddi.13403

20. Byrom, A. E., Craft, M. E., Durant, S. M., Nkwabi, A. J. K., Metzger, K., Hampson, K., . . . Sinclair, A. R. E. (2014). Episodic outbreaks of small mammals influence predator community dynamics in an east African savanna ecosystem [Article]. Oikos, 123(8), 1014-1024. https://doi.org/10.1111/oik.00962

21. Caro, T. M. (1999a). Abundance and distribution of mammals in Katavi National Park, Tanzania [Article]. African Journal of Ecology, 37(3), 305-313. https://doi.org/10.1046/j.1365-2028.1999.00181.x

22. Caro, T. M. (1999b). Densities of mammals in partially protected areas: The Katavi ecosystem of western Tanzania [Article]. Journal of Applied Ecology, 36(2), 205-217. https://doi.org/10.1046/j.1365-2664.1999.00392.x

23. Caro, T. M. (2002). Factors affecting the small mammal community inside and outside Katavi National Park, Tanzania [Article]. Biotropica, 34(2), 310-318. https://doi.org/10.1111/j.1744-7429.2002.tb00542.x

24. Caro, T., Evans, O. S., Fitzherbert, E., Gardner, T. A., Howell, K., Drewes, R., & Shaffer, H. B. (2011). Reptiles of Katavi National Park, Western Tanzania, are from different biomes [Article]. African Journal of Ecology, 49(3), 377-382. https://doi.org/10.1111/j.1365-2028.2011.01261.x

25. Chidodo, S., Kilawe, C. J., Mnyone, L. L., Broecke, B. V., & Mulungu, L. S. (2020). Factors affecting the composition of rodent assemblages in the North Uluguru Mountains, Tanzania. Journal of Vertebrate Biology, 69(2), 20047.20041.
26. Clarke, G. (1995). Status Reports for 6 Coastal Forests in Lindi Region. In: Tanzania.
27. Clarke, G., & Stubblefield, L. (1995). Status Reports for 7 Coastal Forests in Tanga Region, Tanzania. Frontier-Tanzania Technical Report No. 16. The Society for.
28. Cordeiro, N. J., Borghesio, L., Joho, M. P., Monoski, T. J., Mkongewa, V. J., & Dampf, C. J. (2015). Forest fragmentation in an African biodiversity hotspot impacts mixed-species bird flocks [Article]. Biological Conservation, 188, 61-71. https://doi.org/10.1016/j.biocon.2014.09.050
29. Cordeiro, N. J., Lovett, J. C., Mulungu, E., Maina, G. G., & Gerstle, J. H. (2006). Initial trends of bird assemblages before and after river diversion in an endemic-rich African Forest [Article]. Biodiversity and Conservation, 15(3), 971-983. https://doi.org/10.1007/s10531-004-3104-7
30. D'Ammando, G., Caro, T., Oelze, V. M., Phillips, S., Sime, P., Stewart, F. A., & Piel, A. K. (2022). Ecological Drivers of Habitat Use by Meso Mammals in a Miombo Ecosystem in the Issa Valley, Tanzania. Frontiers in Ecology and Evolution, 197.
31. Darbyshire, I., & Ndangalasi, H. J. (2008). Three new species of Barleria sect. Somalia (Acanthaceae) from the miombo woodlands of western Tanzania. Journal of East African Natural History, 97(2), 123-134.
32. de Visser, S. N., Freymann, B. P., Foster, R. F., Nkwabi, A. K., Metzger, K. L., Harvey, A. W., & Sinclair, A. (2015). Invertebrates of the Serengeti: disturbance effects on arthropod diversity and abundance. Serengeti IV Sustain Biodivers Coupled Hum-Nat Syst, 265.
33. Dell, B., Hopkins, A., & Lamont, B. Invertebrate biodiversity of Mkomazi.
34. Doggart, N., Leonard, C., Perkin, A., Menegon, M., & Rovero, F. (2008). The vertebrate biodiversity and forest condition of the North Pare Mountains. TFCG technical paper(17), 1-79.

35. Doggart, N., Perkin, A., Kiure, J., Fjeldså, J., Poynton, J., & Burgess, N. (2006). Changing places: How the results of new field work in the Rubeho Mountains influence conservation priorities in the Eastern Arc Mountains of Tanzania [Review]. African Journal of Ecology, 44(2), 134-144. https://doi.org/10.1111/j.1365-2028.2006.00572.x
36. Doody, K., & Hamerlynck, O. (2003). Biodiversity of Rufiji District—a summary. Unpublished report. Dar es Salaam, Tanzania: Rufiji Environment Management Project.
37. Durant, S. M., Craft, M. M., Foley, C., Hampson, K., Lobora, A. L., Msuha, M., . . . Pettorelli, N. (2010). Does size matter? An investigation of habitat use across a carnivore assemblage in the Serengeti, Tanzania [Article]. Journal of Animal Ecology, 79(5), 1012-1022. https://doi.org/10.1111/j.1365-2656.2010.01717.x
38. Engilis Jr, A., Lalbhai, P. S., & Caro, T. (2009). Avifauna of the Katavi-Rukwa ecosystem, Tanzania. Journal of East African Natural History, 98(1), 95-117.
39. Fitzherbert, E., Gardner, T., Caro, T., & Jenkins, P. (2007). Habitat preferences of small mammals in the Katavi ecosystem of western Tanzania [Article]. African Journal of Ecology, 45(3), 249-257. https://doi.org/10.1111/j.1365-2028.2006.00699.x
40. Fitzherbert, E., Gardner, T., Davenport, T. R. B., & Caro, T. (2006). Butterfly species richness and abundance in the Katavi ecosystem of western Tanzania [Article]. African Journal of Ecology, 44(3), 353-362. https://doi.org/10.1111/j.1365-2028.2006.00655.x
41. Fjeldså, J., Kiure, J., Doggart, N., Hansen, L., & Perkin, A. (2010). Distribution of highland forest birds across a potential dispersal barrier in the Eastern Arc Mountains of Tanzania. Steenstrupia, 32(1), 1-43.
42. Gardner, T. A., Fitzherbert, E. B., Drewes, R. C., Howell, K. M., & Caro, T. (2007). Spatial and temporal patterns of abundance and diversity of an East African leaf litter amphibian fauna. Biotropica, 39(1), 105-113.
43. Gayo, L. (2022). Influence of afforestation on coleopterans abundance and diversity at the University of Dodoma, Tanzania. Environmental and Sustainability Indicators, 16, 100208.
44. Gebert, F. (2022). Mammals and dung beetles along elevational and land use gradients on Mount Kilimanjaro: diversity, traits and ecosystem services Universität Würzburg].

45. Gebrezgiher, G. B., Makundi, R. H., Meheretu, Y., Mulungu, L. S., & Katakweba, A. A. S. (2022). A Decade-Long Change in the Elevational Distribution of Non-Volant Small Mammals on Mount Meru, Tanzania [Article]. Diversity, 14(6), Article 454. https://doi.org/10.3390/d14060454

46. Geeraert, L. (2014). Effects of anthropogenic disturbances on ground beetle (Coleoptera, Carabidae) communities in Afromontane forests: a comparison between habitats with different levels of disturbance in Amani Nature Reserve, Tanzania Norwegian University of Life Sciences, Ås].

47. Giliba, R. A., Boon, E. K., Kayombo, C. J., Musamba, E. B., Kashindye, A. M., & Shayo, P. F. (2011). Species composition, richness and diversity in Miombo woodland of Bereku Forest Reserve, Tanzania. Journal of Biodiversity, 2(1), 1-7.

48. Giliba, R. A., Kaaya, V. S., & Loos, J. Butterfly diversity patterns along a protection gradient in western Tanzania. BIODIVERSITY RESPONSES TO A PROTECTION GRADIENT IN TANZANIA, 33.

49. Gwegime, J., Mwangoka, M., Mulungu, E., Said, H., Latham, J., Gereau, R., . . . es Salaam, D. Two surveys of the biodiversity and forest condition of Rondo Proposed Nature Reserve in 2012 and 2013.

50. Halima, K., & Fraser, G. (2022). Distribution, diversity and abundance of small mammals in different habitat types in the Usangu area, Southern Tanzania. International Journal of Biodiversity and Conservation, 14(3), 139-149.

51. Hall, J. M., Gillespie, T. W., & Mwangoka, M. (2011). Comparison of agroforests and protected forests in the East Usambara Mountains, Tanzania [Article]. Environmental Management, 48(2), 237-247. https://doi.org/10.1007/s00267-010-9579-y

52. Hassan, S. N., & Rija, A. A. (2011). Fire history and management as determinant of patch selection by foraging herbivores in western Serengeti, Tanzania [Article]. International Journal of Biodiversity Science, Ecosystem Services and Management, 7(2), 122-133. https://doi.org/10.1080/21513732.2011.617710

53. Hemp, A. (2002). Ecology of the pteridophytes on the southern slopes of Mt. Kilimanjaro–I. Altitudinal distribution. Plant Ecology, 159, 211-239.

54. Hemp, A. (2006). Continuum or zonation? Altitudinal gradients in the forest vegetation of Mt. Kilimanjaro. Plant Ecology, 184, 27-42.

55. Hemp, A. (2011). 12 Altitudinal zonation and diversity patterns in the forests of Mount Kilimanjaro, Tanzania. Tropical montane cloud forests: science for conservation and management, 134.

56. Hemp, C. (2013). Annotated list of Tettigoniidae (Orthoptera) from the East Usambara Mountains, Tanzania and new Tettigoniidae species from East Africa. Zootaxa, 3737(4), 301-350.

57. Hemp, C., & Heller, K. G. (2019). Orthoptera (Tettigoniidae and Acridoidea) from miombo woodlands of central Tanzania with the description of new taxa [Article]. Zootaxa, 4671(2), 151-194. https://doi.org/10.11646/zootaxa.4671.2.1

58. Howell, K., Msuya, C., & Kihaule, P. (2000). A preliminary biodiversity (fauna) assessment of the Rufiji floodplain and delta. Unpublished report. Dar es Salaam, Tanzania: Rufiji Environment Management Project.

59. Jenkins, R. K. B., Roettcher, K., & Corti, G. (2003). The influence of stand age on wildlife habitat use in exotic teak tree Tectona grandis plantations [Article]. Biodiversity and Conservation, 12(5), 975-990. https://doi.org/10.1023/A:1022877810411

60. Jenkins, R., Corti, G., Fanning, E., & Roettcher, K. (2002). Management implications of antelope habitat use in the Kilombero Valley, Tanzania. Oryx, 36(2), 161-169.

61. Jew, E. K. K., Loos, J., Dougill, A. J., Sallu, S. M., & Benton, T. G. (2015). Butterfly communities in miombo woodland: Biodiversity declines with increasing woodland utilisation [Article]. Biological Conservation, 192, 436-444. https://doi.org/10.1016/j.biocon.2015.10.022

62. John, C. (2018). Assessment of floristic composition, stocking and disturbance in Mkulazi Catchment Forest Reserve in Morogoro District, Tanzania Sokoine University of Agriculture].

63. John, J. R., & Kiwango, H. (2021). Further additions to the avifauna of the isunkaviola plateau, ruaha national park, south-central tanzania, emphasize its ornithological importance [Article]. Scopus: Journal of East African Ornithology, 41(1), 24-34. https://www.scopus.com/inward/record.uri?eid=2-s2.0-85103068898&partnerID=40&md5=2ab72a118d412b6b02f8447c4dcbafd2

64. Jones, T. (2013). Predictors of mammal distribution and abundance in Afromontane forests of the Udzungwa Mountains, Tanzania. Unpublished thesis, Anglia Ruskin University, UK. http://www. stzelephants. org/articles-reports [accessed 6 July 2014].

65. Jones, T., Hawes, J. E., Norton, G. W., & Hawkins, D. M. (2019). Effect of protection status on mammal richness and abundance in Afromontane forests of the Udzungwa Mountains, Tanzania. Biological Conservation, 229, 78-84.

66. Katunzi, T., Soisook, P., Webala, P. W., Armstrong, K. N., & Bumrungsri, S. (2021). Bat activity and species richness in different land-use types in and around Chome Nature Forest Reserve, Tanzania [Article]. African Journal of Ecology, 59(1), 117-131. https://doi.org/10.1111/aje.12783

67. Kavana, P. Y., Sangeda, A. Z., Mtengeti, E. J., Mahonge, C., Bukombe, J., Fyumagwa, R., & Nindi, S. (2019). Herbaceous plant species diversity in communal agro-pastoral and conservation areas in western Serengeti, Tanzania [Article]. Tropical Grasslands-Forrajes Tropicales, 7(5), 502-518. https://doi.org/10.17138/TGFT(7)502-518

68. Kayombo, C. J., Koka, G. E., Mwigune, G., & Kaaya, V. S. (2022). A report on vegetation types, species diversity, and distribution of Monduli mountains forest reserve in Monduli district, northern highlands of Tanzania. Scientific Reports in Life Sciences, 3(2), 15-31.

69. Kayombo, C. J., Lukumay, S., Kaguo, H. J., Kessy, P., & Kahaya, V. (2020). Analysis of Floristic Diversity and Quantification of Diameter Class Aboveground Biomass (AGB) and Carbon Stocks of Afromontane Vegetation of Nkoanenkole Forest Reserve (NCFR) in Northern Tanzania. International Journal of Advanced Research, 2(2), 19-31.

70. Kayombo, C. J., Ndangalasi, H. J., Giliba, R. A., & Kikoti, I. (2022). Assessment of natural regeneration potential of tree species in image forest reserve, Tanzania. International Journal of Advanced Research, 5(1), 49-59.

71. Kiffner, C., Kioko, J., Baylis, J., Beckwith, C., Brunner, C., Burns, C., . . . Kissui, B. (2020). Long-term persistence of wildlife populations in a pastoral area [Article]. Ecology and Evolution, 10(18), 10000-10016. https://doi.org/10.1002/ece3.6658

72. Kikoti, I. A., & Mligo, C. (2015). Impacts of livestock grazing on plant species composition in montane forests on the northern slope of Mount Kilimanjaro, Tanzania [Article]. International Journal of Biodiversity Science, Ecosystem Services and Management, 11(2), 114-127. https://doi.org/10.1080/21513732.2015.1031179

73. Kikoti, I. A., Mligo, C., & Ndangalasi, H. J. (2022). Assessment of plant species composition and natural regeneration in abandoned settlements in the lower montane forest of Kilimanjaro National Park, Tanzania [Article]. Southern Forests, 84(1), 60-69. https://doi.org/10.2989/20702620.2021.2019563
74. Kimaro, J. G. (2007). Changes in vegetation cover and tree biodiversity of wetland ecosystems of Ngumburuni Forest Reserve, Rufiji District, Coast Region, Tanzania Sokoine University of Agriculture (SUA)].
75. Kisingo, A. W., Sabuni, C. A., Coiffait, L., Hayhow, B., & Larsen, B. (2005). Effects of habitat fragmentation on diversity of small mammals in Lulanda Forest in Mufindi, Tanzania [Article]. Belgian Journal of Zoology, 135(SUPPL.1), 109-112. https://www.scopus.com/inward/record.uri?eid=2-s2.0-53549096852&partnerID=40&md5=8d5c9a31fd540ac42708607680df3080
76. Kiwia, H. (2006). Species richness and abundance estimates of small mammals in Zaraninge coastal forest in Tanzania. Tanzania Journal of Science, 32(2), 51-60.
77. Krüger, O., & MCGAVIN, G. (1997). The insect fauna of Acacia species in Mkomazi Game Reserve, north-east Tanzania. Ecological Entomology, 22(4), 440-444.
78. Kunene, C. (2020). Ant communities along an elevational transect, the Udzungwa Mountains in Tanzania
79. Kunene, C., Foord, S. H., Scharff, N., Pape, T., Malumbres-Olarte, J., & Munyai, T. C. (2022). Ant diversity declines with increasing elevation along the Udzungwa Mountains, Tanzania. Diversity, 14(4), 260.
80. Lasway, J. V., Kinabo, N. R., Mremi, R. F., Martin, E. H., Nyakunga, O. C., Sanya, J. J., . . . Njovu, H. K. (2021). A synopsis of the Bee occurrence data of northern Tanzania [Article]. Biodiversity Data Journal, 9, 1-16. https://doi.org/10.3897/BDJ.9.e68190
81. Lasway, J. V., Njovu, H. K., Eustace, A., Mathisen, K. M., Skarpe, C., & Peters, M. K. (2023). Species richness, vegetation structure, and floristic composition of woody plants along the elevation gradient of Mt. Meru, Tanzania [Article]. Biotropica. https://doi.org/10.1111/btp.13232

82. Lasway, J. V., Steffan-Dewenter, I., Njovu, H. K., Kinabo, N. R., Eardley, C., Pauly, A., & Peters, M. K. (2022). Positive effects of low grazing intensity on East African bee assemblages mediated by increases in floral resources [Article]. Biological Conservation, 267, Article 109490. https://doi.org/10.1016/j.biocon.2022.109490
83. Lawson, L. P., Loader, S. P., Lyakurwa, J. V., & Liedtke, H. C. (2023). Diversification of spiny-throated reed frogs (Anura: Hyperoliidae) with the description of a new, range-restricted species from the Ukaguru Mountains, Tanzania [Article]. PLoS ONE, 18(2 February), Article e0277535. https://doi.org/10.1371/journal.pone.0277535
84. Lawson, L., & Moyer, D. (2008). Within and between-site distribution of frog species on the Udzungwa Plateau, Tanzania. African Journal of Herpetology, 57(2), 93-102.
85. Lema, R., & Magige, F. J. (2018). The influence of agricultural activities on the diversity of rodents in Kindoroko forest reserve and surrounding areas, North Pare Mountains, Tanzania. Tanzania Journal of Science, 44(1), 97-106.
86. Levi, M., Lee, D. E., Bond, M. L., & Treydte, A. C. (2022). Forage selection by Masai giraffes (Giraffa camelopardalis tippelskirchi) at multiple spatial scales. Journal of Mammalogy, 103(3), 737-744.
87. Liedtke, H. C., Lyakurwa, J. V., Lawson, L. P., Menegon, M., Garrido-Priego, M., Mariaux, J., . . . Loader, S. P. (2022). Thirty years of amphibian surveys in the Ukagurus Mountains of Tanzania reveal new species, yet others are in decline [Article]. African Journal of Herpetology, 71(2), 119-138. https://doi.org/10.1080/21564574.2022.2043945
88. Liseki, S. D., & Vane-Wright, R. I. (2011). Butterflies (Lepidoptera: Papilionoidea) of mount kilimanjaro: Introduction and family papilionidae [Article]. Journal of Natural History, 45(37-38), 2375-2396. https://doi.org/10.1080/00222933.2011.596635
89. Loftis, E. E. (2015). Population Viability of Mega-Herbivores in Manyara Ranch, Tanzania, in a Climate Change Context.
90. Lovett, J. C. (1996). Elevational and latitudinal changes in tree associations and diversity in the Eastern Arc mountains of Tanzania. Journal of Tropical Ecology, 12(5), 629-650.
91. Lovett, J. C., Clarke, G. P., Moore, R., & Morrey, G. H. (2001). Elevational distribution of restricted range forest tree taxa in eastern Tanzania. Biodiversity & Conservation, 10, 541-550.

92. Lovett, J. C., Marshall, A. R., & Carr, J. (2006). Changes in tropical forest vegetation along an altitudinal gradient in the Udzungwa Mountains National Park, Tanzania. African Journal of Ecology, 44(4), 478-490.

93. Lyakurwa, J. V., Howell, K. M., Munishi, L. K., & Treydte, A. C. (2019). Uzungwa scarp nature forest reserve: A unique hotspot for reptiles in Tanzania [Article]. Acta Herpetologica, 14(1), 3-14. https://doi.org/10.13128/Acta_Herpetol-25008

94. Lyaruu, H. V., Eliapenda, S., & Backéus, I. (2000). Floristic, structural and seed bank diversity of a dry Afromontane forest at Mafai, central Tanzania. Biodiversity & Conservation, 9, 241-263.

95. Lyimo, P., & Shaaban, S. (2015). Assessment of Forest condition at SUA-Kitulangalo forest reserve in Tanzania Miombo Woodland. GRIN Verlag.

96. Magige, F. J. (2013). Rodent species diversity in relation to altitudinal gradient in Northern Serengeti, Tanzania [Article]. African Journal of Ecology, 51(4), 618-624. https://doi.org/10.1111/aje.12075

97. Maliondo, S. M. S., Abeli, W. S., Meiludie, R. E. L. O., Migunga, G. A., Kimaro, A. A., & Applegate, G. B. (2005). Tree species composition and potential timber production of a communal miombo woodland in Handeni district, Tanzania [Article]. Journal of Tropical Forest Science, 17(1), 104-120. https://www.scopus.com/inward/record.uri?eid=2-s2.0-16244392398&partnerID=40&md5=f54979fa667fee6b50663857eecce71d

98. Mangora, M. M., Lugendo, B. R., Shalli, M. S., & Semesi, S. (2016). Mangroves of Tanzania. Mangroves of the Western Indian Ocean: status and management, 33-49.

99. Manyonyi, A. M., Mariki, S. B., Mnyone, L. L., Belmain, S. R., & Mulungu, L. S. (2020). Effects of prescribed burning on rodent community ecology in Serengeti National Park [Article]. Journal of Vertebrate Biology, 69(2), Article 20001. https://doi.org/10.25225/jvb.20001

100. Mapunda, R., & John, J. R. (2021). Effects of wildfire on vegetation and understory avian communities in montane rainforests, north-eastern Tanzania [Article]. African Journal of Ecology, 59(2), 466-478. https://doi.org/10.1111/aje.12831

101. Marshall, A. R. (2007). Assessing and Restoring Biodiversity in Tanzania's Forests. The Case of Magombera. Proceedings of the 6th TAWIRI Scientific conference,

102. Masikini, R., Kaaya, L. T., & Chicharo, L. (2018). Evaluation of ecohydrological variables in relation to spatial and temporal variability of macroinvertebrate assemblages along the Zigi River–Tanzania. Ecohydrology & Hydrobiology, 18(2), 130-141.

103. Mattila, P., & Koponen, T. (1999). Diversity of bryophyte flora and vegetation on rotten wood in rain and montane forests of northeastern Tanzania. Tropical Bryology, 139-164.

104. Mbije, N. E., & Kamungu, A. (2021). Rainy Season Food Availability for Anurans of Kimboza Forest Reserve, Tanzania. Asian Journal of Biology, 12(4), 15-25.

105. Mduma, S. A., Sinclair, A., & Turkington, R. (2007). The role of rainfall and predators in determining synchrony in reproduction of savanna trees in Serengeti National Park, Tanzania. Journal of Ecology, 95(1), 184-196.

106. Menegon, M., Bracebridge, C., Owen, N., & Loader, S. P. (2011). Herpetofauna of montane areas of Tanzania. 4. Amphibians and reptiles of Mahenge Mountains, with comments on biogeography, diversity, and conservation. Fieldiana Life and Earth Sciences, 2011(4), 103-111.

107. Menegon, M., Lyakurwa, J. V., Loader, S. P., & Tolley, K. A. (2022). Cryptic diversity in pygmy chameleons (Chamaeleonidae: Rhampholeon) of the Eastern Arc Mountains of Tanzania, with description of six new species [Article]. Acta Herpetologica, 17(2), 85-113. https://doi.org/10.36253/a_h-12978

108. Menegon, M., Salvidio, S., & Tilbury, C. (2002). A new dwarf forest chameleon from the Udzungwa Mountains of Tanzania, East Africa,(Squamata: Rhampholeon Günther, 1874). Journal of Herpetology, 51-57.

109. Mercader, J., Clarke, S., Bundala, M., Favreau, J., Inwood, J., Itambu, M., . . . Mollel, N. (2019). Soil and plant phytoliths from the Acacia-Commiphora mosaics at Oldupai Gorge (Tanzania). PeerJ, 7, e8211.

110. Mgelwa, A., Mpita, M., Rija, A., Kabalika, Z., & Hassan, S. (2023). Avifauna community in a threatened conservation landscape, western Tanzania: a baseline. Tanzania Journal of Forestry and Nature Conservation, 92(1), 10-24.

111. Mkonyi, F. J. (2021). Biodiversity hotspot revisited: reptile and amphibian assemblages of the Uluguru Mountain Forest Reserves, south-eastern Tanzania. African Journal of Herpetology, 70(2), 95-122.

112. Mligo, C. (2015). Conservation of plant biodiversity of Namatimbili forest in the southern coastal forests of Tanzania. International Journal of Biodiversity and Conservation, 7(3), 148-172.

113. Mligo, C. (2015). Plant species composition and distribution in relation to land use patterns in serengeti ecosystem Tanzania. Open Journal of Forestry, 5(06), 607.

114. Mligo, C. (2017). Diversity and distribution pattern of riparian plant species in the Wami River system, Tanzania. Journal of Plant Ecology, 10(2), 259-270.

115. Mligo, C. (2018). The plant species composition, diversity and natural regeneration of indigenous trees in the disturbed Ruvu South Forest Reserve, Tanzania. Tanzania Journal of Science, 44(3), 46-60.

116. Mligo, C. (2019). Post fire regeneration of indigenous plant species in the Pugu Forest Reserve, Tanzania. Global Ecology and Conservation, 18, e00611.

117. Mligo, C., Lyaruu, H., Ndangalasi, H., & Marchant, R. (2009). Vegetation community structure, composition and distribution pattern in the Zaraninge Forest, Bagamoyo District, Tanzania. Journal of East African Natural History, 98(2), 223-239.

118. Modest, R. B., & Hassan, S. N. (2016). Species composition of tropical understory birds in threatened east African coastal forests based on capture data. International Journal of Zoology, 2016.

119. Moehlman, P. D., Ogutu, J. O., Piepho, H. P., Runyoro, V. A., Coughenour, M. B., & Boone, R. B. (2020). Long-term historical and projected herbivore population dynamics in Ngorongoro crater, Tanzania [Article]. PLoS ONE, 15(3), Article e0212530. https://doi.org/10.1371/journal.pone.0212530

120. Mohamed, N. S. (2023). Seasonal and Periodical Assessment of the Abundance and Diversity of Epigaeic Invertebrates in an Urban Forest Remnant, Dar es Salaam, Tanzania. East African Journal of Environment and Natural Resources, 6(1), 117-135.

121. Mollel, N. P., Fischer, M., & Hemp, A. (2017). Usable wild plant species in relation to elevation and land use at Mount Kilimanjaro, Tanzania [Article]. Alpine Botany, 127(2), 145-154. https://doi.org/10.1007/s00035-017-0187-9

122. Mpondo, F. T., Ndakidemi, P. A., Pauly, A., & Treydte, A. C. (2021). Traditional rangeland management can conserve insect pollinators in a semi-arid rangeland, northern Tanzania [Article]. Acta Oecologica, 113, Article 103790. https://doi.org/10.1016/j.actao.2021.103790

123. Mseja, G. A., Furael, B. B., Lyakurwa, G. J., & Martin, E. H. (2020). Herbaceous vegetation communities around lake manyara, tanzania: Response to environmental gradients [Article]. Nature Conservation Research, 5(4), 55-64. https://doi.org/10.24189/ncr.2020.056

124. Msindai, I. M. (2014). An assessment of diversity, abundance and distribution of herpetofauna in the Serengeti National Park, Tanzania Sokoine University of Agriculture].

125. Msuya, T. S., & Kideghesho, J. R. (2009). The role of traditional management practices in enhancing sustainable use and conservation of medicinal plants in West Usambara Mountains, Tanzania. Tropical Conservation Science, 2(1), 88-105.

126. Mtui, D., Owen-Smith, N., & Lepczyk, C. (2017). Assessment of wildlife populations trends in three protected areas in Tanzania from 1991 to 2012 [Article]. African Journal of Ecology, 55(3), 305-315. https://doi.org/10.1111/aje.12354

127. Mulungu, L. S., Makundi, R. H., Massawe, A. W., Machang'u, R. S., & Mbije, N. E. (2008). Diversity and distribution of rodent and shrew species associated with variations in altitude on Mount Kilimanjaro, Tanzania [Conference Paper]. Mammalia, 72(3), 178-185. https://doi.org/10.1515/MAMM.2008.021

128. Mungai, F., Kairo, J., Mironga, J., Kirui, B., Mangora, M., & Koedam, N. (2019). Mangrove cover and cover change analysis in the transboundary area of Kenya and Tanzania during 1986–2016. Journal of the Indian Ocean Region, 15(2), 157-176.

129. Munishi, P. K., Temu, R.-A. P., & Soka, G. (2011). Plant communities and tree species associations in a Miombo ecosystem in the Lake Rukwa basin, Southern Tanzania: Implications for conservation.

130. Munishi, P., Shear, T., Wentworth, T., Temu, R., & Maliondo, S. (2004). Sparse Distribution Pattern Of Some Plant Species In Two Afromontane Rain Forests Of The Eastern Arc Mountains Of Tanzania. Tanzania journal of forestry and nature conservation, 75(1), 74-90.

131. Musese, L. J., Andrew, S. M., Shirima, D. D., Witt, A., & Kilewa, R. (2020). Effects of the abundance of Parthenium hysterophorus on the composition and diversity of other herbaceous plant species in Simanjiro Rangeland, Tanzania.

132. MWAKALUKWA, E. E., MWAKISU, A., & MALIONDO, S. M. S. (2023). Woody species diversity, composition, structure and carbon storage of a dry evergreen montane forest of Essimingor Nature Forest Reserve in Tanzania. International Journal of Tropical Drylands, 7(1).

133. Mwaluseke, M. L., Mwakalukwa, E. E., & Maliondo, S. M. S. (2023). Vegetation composition, diversity, stand structure and carbon stock of a dry evergreen montane forest of Lendikinya forest reserve in Tanzania [Article]. Biodiversitas, 24(1), 551-562. https://doi.org/10.13057/biodiv/d240164

134. Mwasumbi, L., Burgess, N., & Clarke, G. (1994). Vegetation of Pande and Kiono coastal forests, Tanzania. Vegetatio, 113, 71-81.

135. Mwasumbi, L., Suleiman, H., & Lyaruu, V. (2000). A Preliminary Biodiversity (Flora) Assessment of Selected Forests of the Rufiji Floodplain. Rufiji Environment Management Project, Dar es Salaam, Tanzania.

136. Mwendwa, B. A., Kaaya, O. E., Kilawe, C. J., & Treydte, A. C. (2020). Spatio-temporal invasion dynamics of Maesopsis eminii in Amani Nature Forest Reserve, Tanzania [Article]. Forest Ecology and Management, 465, Article 118102. https://doi.org/10.1016/j.foreco.2020.118102

137. Nasseri, N. A. (2009). The relationship of herpetofaunal community composition to an elephant (Loxodonta africana) modified savanna woodland of northern Tanzania, and bioassays with African elephants.

138. Nehemia, A., Chen, M., Kochzius, M., Dehairs, F., & Brion, N. (2019). Ecological impact of salt farming in mangroves on the habitat and food sources of Austruca occidentalis and Littoraria subvittata. Journal of Sea Research, 146, 24-32.

139. Nehemia, A., Ngendu, Y., & Kochzius, M. (2019). Genetic population structure of the mangrove snails Littoraria subvittata and L. pallescens in the Western Indian Ocean. Journal of Experimental Marine Biology and Ecology, 514, 27-33.

140. Njana, M. A. (2020). Structure, growth, and sustainability of mangrove forests of mainland Tanzania. Global Ecology and Conservation, 24, e01394.

141. Njana, M. A., Zahabu, E., & Malimbwi, R. E. (2018). Carbon stocks and productivity of mangrove forests in Tanzania. Southern Forests: a Journal of Forest Science, 80(3), 217-232.

142. Nkwabi, A. K., Lyamuya, R. D., Masenga, E., Bukombe, J., Mwakalebe, G., Mdaki, M., & Fyumagwa, R. (2018). Spatial-temporal distribution, abundance, diversity and mortality of birds on road network in the Serengeti Ecosystem, Tanzania. International Journal of Biodiversity and Conservation, 10(4), 192-202.

143. Notø, C. (2014). Effects of anthropogenic disturbance of Afromontane forest on butterflies (Lepidoptera, Nymphalidae) in Amani Nature Reserve, Tanzania Norwegian University of Life Sciences, Ås].

144. Ntukey, L. T., Munishi, L. K., & Treydte, A. C. (2022). Land Use Land/Cover Change Reduces Woody Plant Diversity and Carbon Stocks in a Lowland Coastal Forest Ecosystem, Tanzania. Sustainability, 14(14), 8551.

145. Nyomora, A. (2005). Distribution and abundance of the edible orchids of the Southern Highlands of Tanzania. Tanzania Journal of Science, 31(1), 45-54.

146. Ojoyi, M., Mutanga, O., Odindi, J., Aynekulu, E., & Abdel-Rahman, E. (2015). The effect of forest fragmentation on tree species abundance and diversity in the Eastern Arc Mountains of Tanzania. Appl. Ecol. Environ. Res, 13(2), 307-324.

147. Piel, A. K., Bonnin, N., RamirezAmaya, S., Wondra, E., & Stewart, F. A. (2019). Chimpanzees and their mammalian sympatriates in the Issa Valley, Tanzania [Article]. African Journal of Ecology, 57(1), 31-40. https://doi.org/10.1111/aje.12570

148. Renner, M., Rembold, K., Hemp, A., & Fischer, M. (2022). Natural regeneration of woody plant species along an elevational and disturbance gradient at Mt. Kilimanjaro. Forest Ecology and Management, 520, 120404.

149. Richard, U., Byamungu, R. M., Magige, F., & Makonda, F. B. S. (2022). Microhabitat, altitude and seasonal influence on the abundance of non-volant small mammals in Mount Rungwe forest nature reserve [Article]. Global Ecology and Conservation, 35, Article e02069. https://doi.org/10.1016/j.gecco.2022.e02069

150. Rija, A. A. (2022). Local habitat characteristics determine butterfly diversity and community structure in a threatened Kihansi gorge forest, Southern Udzungwa Mountains, Tanzania [Article]. Ecological Processes, 11(1), Article 13. https://doi.org/10.1186/s13717-022-00359-z

151. Rija, A. A., Mgelwa, A. S., Modest, R. B., & Hassan, S. N. (2015). Composition and functional diversity in bird communities in a protected humid coastal savanna. Advances in Zoology, 2015.

152. Robertson, H. G. (2002). Comparison of leaf litter ant communities in woodlands, lowland forests and montane forests of north-eastern Tanzania. Biodiversity & Conservation, 11, 1637-1652.

153. Rossi, R., Barocco, R., Salvidio, S., & Menegon, M. (2010). Montane grasslands of the Udzungwa plateau, Tanzania: A study case about its herpetological importance within the Eastern Afromontane hotspot. In Grassland Biodiversity: Habitat Types, Ecological Processes and Environmental Impacts (pp. 179-200). Nova Science Publishers, Inc. https://www.scopus.com/inward/record.uri?eid=2-s2.0-85048664263&partnerID=40&md5=ba45c0f33e5aabc50f694b1b3c03148b

154. Rovero, F., Marshall, A. R., Jones, T., & Perkin, A. (2009). The primates of the Udzungwa Mountains: diversity, ecology and conservation. Journal of Anthropological Sciences= Rivista di Antropologia: JASS, 87, 93-126.

155. Rovero, F., Martin, E., Rosa, M., Ahumada, J. A., & Spitale, D. (2014). Estimating species richness and modelling habitat preferences of tropical forest mammals from camera trap data [Article]. PLoS ONE, 9(7), Article e103300. https://doi.org/10.1371/journal.pone.0103300

156. Rovero, F., Menegon, M., Fjeldså, J., Collett, L., Doggart, N., Leonard, C., . . . Burgess, N. D. (2014). Targeted vertebrate surveys enhance the faunal importance and improve explanatory models within the Eastern Arc Mountains of Kenya and Tanzania [Article]. Diversity and Distributions, 20(12), 1438-1449. https://doi.org/10.1111/ddi.12246

157. Rovero, F., Rathbun, G., Perkin, A., Jones, T., Ribble, D. O., Leonard, C., . . . Doggart, N. (2008). A new species of giant sengi or elephant-shrew (genus Rhynchocyon) highlights the exceptional biodiversity of the Udzungwa Mountains of Tanzania. Journal of Zoology, 274(2), 126-133.

158. Rumisha, C., Mdegela, R. H., Gwakisa, P. S., & Kochzius, M. (2018). Genetic diversity and gene flow among the giant mud crabs (Scylla serrata) in anthropogenic-polluted mangroves of mainland Tanzania: implications for conservation. Fisheries Research, 205, 96-104.

159. Rumisha, C., Shukuru, H., Lyimo, J., Maganira, J. D., & Nehemia, A. (2015). Benthic macroinvertebrate assemblages in mangroves and open intertidal areas on the Dar es Salaam coast, Tanzania. African Journal of Aquatic Science, 40(2), 143-151.

160. Russell-Smith, A. (2002). A comparison of the diversity and composition of ground-active spiders in Mkomazi Game Reserve, Tanzania and Etosha National Park, Namibia. The Journal of Arachnology, 30(2), 383-388.

161. Ruvuga, P. R., Wredle, E., Nyberg, G., Hussein, R. A., Masao, C. A., Selemani, I. S., . . . Kronqvist, C. (2021). Evaluation of rangeland condition in miombo woodlands in eastern Tanzania in relation to season and distance from settlements [Article]. Journal of Environmental Management, 290, Article 112635. https://doi.org/10.1016/j.jenvman.2021.112635

162. Saanya, A., Mulungu, L., Sabuni, C., Massawe, A., & Makundi, R. (2023). Effects of prescribed burning on rodents in an East African woodland ecosystem [Article]. African Journal of Ecology. https://doi.org/10.1111/aje.13143

163. Sabuni, C. A., Sluydts, V., Mulungu, L. S., Maganga, S. L., Makundi, R. H., & Leirs, H. (2015). Distribution and ecology of lesser pouched rat, Beamys hindei, in Tanzanian coastal forests. Integrative Zoology, 10(6), 531-542.

164. Sabuni, C., Aghová, T., Bryjová, A., Šumbera, R., & Bryja, J. (2018). Biogeographic implications of small mammals from Northern Highlands in Tanzania with first data from the volcanic Mount Kitumbeine. Mammalia, 82(4), 360-372.

165. Sabuni, C., Van Houtte, N., Gryseels, S., Maganga, S., Makundi, R. H., Leirs, H., & Goüy de Bellocq, J. (2016). Genetic structure and diversity of the black and rufous sengi in Tanzanian coastal forests. Journal of Zoology, 300(4), 305-313.

166. Sæbjørnsen, M. (2016). Impact of Anthropogenic Forest Disturbance on Dung Beetle (Coleoptera, Scarabaeidae) Communities in Amani Nature Reserve Norwegian University of Life Sciences, Ås].

167. Sangiwa, M. W., & Magige, F. J. (2019). Effects of roads on small mammal diversity and abundance in the northern Serengeti, Tanzania [Article]. African Journal of Ecology, 57(4), 565-574. https://doi.org/10.1111/aje.12637

168. Seddon, N., Ekstrom, J. M. M., Capper, D. R., Isherwood, I. S., Muna, R., Pople, R. G., . . . Timothy, J. (1999). Notes on the ecology and conservation status of key bird species in Nilo and Nguu North Forest Reserves, Tanzania [Article]. Bird Conservation International, 9(1), 9-23. https://doi.org/10.1017/s0959270900003312

169. Seki, H. A., Shirima, D. D., Courtney Mustaphi, C. J., Marchant, R., & Munishi, P. K. (2018). The impact of land use and land cover change on biodiversity within and adjacent to Kibasira Swamp in Kilombero Valley, Tanzania. African Journal of Ecology, 56(3), 518-527.

170. Semesi, A. K. (1992). Developing management plans for the mangrove forest reserves of mainland Tanzania. The Ecology of Mangrove and Related Ecosystems: Proceedings of the International Symposium held at Mombasa, Kenya, 24–30 September 1990,

171. Shangali, C., Mabula, C., & Mmari, C. (1998). Biodiversity and human activities in the Udzungwa Mountain forests, Tanzania. 1. Ethnobotanical survey in the Uzungwa Scarp Forest Reserve. Journal of East African Natural History, 87(1), 291-318.

172. Shilereyo, M. T., Magige, F. J., Ogutu, J. O., & Røskaft, E. (2021). Land use and habitat selection by small mammals in the Tanzanian Greater Serengeti Ecosystem [Article]. Global Ecology and Conservation, 27, Article e01606. https://doi.org/10.1016/j.gecco.2021.e01606

173. Shirima, D. D., Totland, O., Munishi, P. K. T., & Moe, S. R. (2015). Does the abundance of dominant trees affect diversity of a widespread tropical woodland ecosystem in Tanzania? [Article]. Journal of Tropical Ecology, 31(4), 345-359. https://doi.org/10.1017/S0266467415000231

174. Sinclair, A. R. E., Mduma, S. A. R., & Arcese, P. (2002). Protected areas as biodiversity benchmarks for human impact: Agriculture and the Serengeti avifauna [Article]. Proceedings of the Royal Society B: Biological Sciences, 269(1508), 2401-2405. https://doi.org/10.1098/rspb.2002.2116
175. Sitati, N., Gichohi, N., Lenaiyasa, P., Millanga, P., Maina, M., Warinwa, F., & Muruthi, P. (2014). Tre e species diversity and dominance in Gelai Forest Reserve, Tanzania. Journa l of Energy and Natural Resources, 3, 31-37.
176. Smith, A. R., & Jocqué, R. (2015). New Zodariidae (Araneae) from Mkomazi Game Reserve, Tanzania. African Invertebrates, 56(2), 455-476.
177. Soka, G. E., Munishi, P. K., & Thomas, M. B. (2013). Species diversity and abundance of Avifauna in and around Hombolo Wetland in Central Tanzania. Int. J. Biodivers. Conserv, 5(11), 782-790.
178. Sørensen, L. (1993). Section 7: Invertebrates, with the main emphasis placed on millipedes. Report on the Uluguru Biodiversity Survey, 60-63.
179. Sørensen, L. L. (2004). Composition and diversity of the spider fauna in the canopy of a montane forest in Tanzania. Biodiversity & Conservation, 13, 437-452.
180. Sørensen, L. L., Coddington, J. A., & Scharff, N. (2002). Inventorying and estimating subcanopy spider diversity using semiquantitative sampling methods in an Afromontane forest. Environmental Entomology, 31(2), 319-330.
181. Stanbrook, R., Norrey, J., Kisingo, A. W., & Jones, M. (2021). Dung beetle diversity and community composition along a land use gradient in a Savannah Ecosystem of North Western Tanzania. Tropical Conservation Science, 14, 19400829211008756.
182. Stanley, W. T. (2015). Elevational Distribution and Taxonomy of Shrews and Rodents in the Mountains of Northern Tanzania Staats-und Universitätsbibliothek Hamburg Carl von Ossietzky].
183. Stanley, W. T., & Kihaule, P. M. (2016). Elevational distribution and ecology of small mammals on Tanzania's second highest mountain [Article]. PLoS ONE, 11(9), Article e0162009. https://doi.org/10.1371/journal.pone.0162009

184. Stanley, W. T., Goodman, S. M., & Newmark, W. D. (2011). Small mammal inventories in the East and West Usambara Mountains, Tanzania. 1. Study areas, methodologies, and general results. Fieldiana Life and Earth Sciences, 2011(4), 1-17.
185. Stanley, W. T., Kihaule, P. M., & Munissi, M. J. (2007). Small mammals of two forest reserves in the North Pare Mountains, Tanzania. Journal of East African Natural History, 96(2), 215-226.
186. Stanley, W. T., Rogers, M. A., Howell, K. M., & Msuya, C. A. (2005). Results of a survey of small mammals in the Kwamgumi Forest Reserve, East Usambara Mountains, Tanzania. Journal of East African Natural History, 94(1), 223-230.
187. Stanley, W. T., Rogers, M. A., Kihaule, P. M., & Munissi, M. J. (2014). Elevational distribution and ecology of small mammals on Africa's highest mountain. PLoS One, 9(11), e109904.
188. Tanzania, W., Howell, K., Msuya, C., Mligo, C., Werema, C., Kihaule, P., . . . Suleiman, H. (2012). Biodiversity Surveys of Poorly Known Coastal Forests of Southeastern Tanzania and Zanzibar. Fieldwork, Tanzania.
189. Temu, R., & Andrew, S. (2008). Endemism of plants in the Uluguru Mountains, Morogoro, Tanzania. Forest Ecology and Management, 255(7), 2858-2869.
190. Thomas, S. M., Soka, G. E., & Mulungu, L. S. (2022). Influence of vegetation structure, seasonality, and soil properties on rodent diversity and community assemblages in west Mount Kilimanjaro, Tanzania [Article]. Ecology and Evolution, 12(9), Article e9211. https://doi.org/10.1002/ece3.9211
191. Thomas, S. M., Soka, G. E., Mulungu, L. S., & Makonda, F. B. (2022). Spatial-temporal variations in dietary consumption of two dominant rodent species (Rhabdomys dilectus and Lophuromys acquilus) on Mount Kilimanjaro, Tanzania. Diversity, 14(8), 659.
192. Timbuka, C., & Kabigumila, J. (2006). Diversity and abundance of small mammals in the Serengeti Kopjes, Tanzania. Tanzania Journal of Science, 32(1), 1-12.
193. Torres, P., Alfiado, A., Glassom, D., Jiddawi, N., Macia, A., Reid, D. G., & Paula, J. (2008). Species composition, comparative size and abundance of the genus Littoraria (Gastropoda: Littorinidae) from different mangrove strata along the East African coast. Hydrobiologia, 614, 339-351.

194. Trentin, L., & Rovero, F. (2011). Results of an inventory of bats in the Uzungwa scarp forest reserve, Tanzania. Journal of East African Natural History, 100(1&2), 45-57.
195. Vihemäki, H., Hall, J. M., Leonard, C., Mwangoka, M., & Mkongewa, V. (2013). Bird and plant diversity in tropical landscape mosaics in East Usambaras, Tanzania. Small-scale Forestry, 12, 125-143.
196. Villet, M. H., Van Noort, S., & Packer, M. (1999). Cicadas (Hemiptera, Homoptera: Cicadoidea) of Mkomazi. Mkomazi: The Ecology, Biodiversity and Conservation of a Tanzania savanna. Royal Geographical Society (with the Institute of British Geographers), London, 223-234.
197. Wagner, G., Akwilapo, F., Mrosso, S., Ulomi, S., & Masinde, R. (2004). Assessment of Marine Biodiversity, Ecosystem Health, and Resource Status in Mangrove Forests in Mnazi Bay Ruvuma Estuary Marine Park, viii+ 106pp. UNDP/GEF and FFEM.
198. Waltert, M., Meyer, B., Shanyangi, M. W., Balozi, J. J., Kitwara, O., Qolli, S., . . . Muhlenberg, M. (2008). Foot surveys of large mammals in woodlands of western Tanzania [Article]. Journal of Wildlife Management, 72(3), 603-610. https://doi.org/10.2193/2006-456
199. Washa, B. W. (2023). Plant Species Composition and Diversity in Dalbergia melanoxylon Dominated Zones in Mitarure Forest Reserve in Kilwa District.
200. Werema, C. (2021). Bird species diversity and community composition in four woodland types in the Serengeti ecosystem, Tanzania [Article]. African Journal of Ecology, 59(3), 760-768. https://doi.org/10.1111/aje.12857
201. Werema, C. (2021). The avifauna of a naturally regenerating secondary forest, Pangani, North-Eastern Tanzania. Journal of East African Natural History, 110(2), 77–85-77–85.
202. Werema, C., & Howell, K. M. (2016). Seasonal variation in diversity and abundance of understorey birds in Bunduki Forest Reserve, Tanzania: evaluating the conservation value of a plantation forest. Ostrich, 87(1), 89-93.

203. Werema, C., & Nahonyo, C. L. (2022). Further surveys of the miombo woodland avifaunas of Mbarang'andu and Kimbanda Wildlife Management Areas, southern Tanzania [Note]. Scopus: Journal of East African Ornithology, 42(2), 38-50. https://www.scopus.com/inward/record.uri?eid=2-s2.0-85135601681&partnerID=40&md5=fb9d337bb53a6eb713513886b0c254d8

204. Werema, C., McEntee, J. P., Mulungu, E., & Mbilinyi, M. (2012). Preliminary observations on the avifauna of Ikokoto Forest, Udzungwa Mountains, Tanzania. Scopus: Journal of East African Ornithology, 32, 19–26-19–26.

205. Wesolowska, W., & Russell-Smith, A. (2000). Jumping spiders from Mkomazi Game Reserve in Tanzania (Araneae Salticidae). Tropical zoology, 13(1), 11-127.

206. Williams, E. V., Ntandu, J. E., Ficinski, P., & Vorontsova, M. (2016). Checklist of Serengeti Ecosystem Grasses [Article]. Biodiversity Data Journal, 4(1), Article e8286. https://doi.org/10.3897/BDJ.4.e8286

207. Zilihona, I. J., & Nummelin, M. (2001). Coleopteran diversity and abundance in different habitats near Kihansi waterfall, in the Udzungwa Mountains, Tanzania. Biodiversity & Conservation, 10, 769-777.

208. Zilihona, I. J., Niemelä, J., & Nummelin, M. (2004). Effects of a hydropower plant on Coleopteran diversity and abundance in the Udzungwa Mountains, Tanzania. Biodiversity & Conservation, 13, 1453-1464.
